# Development of Novel Isatin-Tethered Quinolines as Anti-Tubercular Agents against Multi and Extensively Drug-Resistant *Mycobacterium tuberculosis*

**DOI:** 10.3390/molecules27248807

**Published:** 2022-12-12

**Authors:** Mohamed A. Abdelrahman, Hadia Almahli, Tarfah Al-Warhi, Taghreed A. Majrashi, Marwa M. Abdel-Aziz, Wagdy M. Eldehna, Mohamed A. Said

**Affiliations:** 1 Department of Pharmaceutical Chemistry, Faculty of Pharmacy, Egyptian Russian University, Badr City 11829, Egypt; 2 Department of Chemistry, University of Cambridge, Cambridge CB2 1EW, UK; 3 Department of Chemistry, College of Science, Princess Nourah bint Abdulrahman University, P.O. Box 84428, Riyadh 11671, Saudi Arabia; 4 Department of Pharmacognosy, College of Pharmacy, King Khalid University, Abha 61441, Saudi Arabia; 5 The Regional Center for Mycology and Biotechnology, Al-Azhar University, Cairo 11651, Egypt; 6 Department of Pharmaceutical Chemistry, Faculty of Pharmacy, Kafrelsheikh University, Kafrelsheikh 33516, Egypt; 7 School of Biotechnology, Badr University in Cairo, Cairo 11829, Egypt

**Keywords:** quinolone, hydrazone, anti-mycobacterial activity, *Mycobacterium* resistance

## Abstract

We describe the design and synthesis of two isatin-tethered quinolines series (**Q6a**–**h** and **Q8a**–**h**), in connection with our research interest in developing novel isatin-bearing anti-tubercular candidates. In a previous study, a series of small molecules bearing a quinoline-3-carbohydrazone moiety was developed as anti-tubercular agents, and compound **IV** disclosed the highest potency with MIC value equal to 6.24 µg/mL. In the current work, we adopted the bioisosteric replacement approach to replace the 3,4,5-trimethoxy-benzylidene moiety in the lead compound **IV** with the isatin motif, a privileged scaffold in the TB drug discovery, to furnish the first series of target molecules **Q6a**–**h**. Thereafter, the isatin motif was *N*-substituted with either a methyl or benzyl group to furnish the second series **Q8a**–**h**. All of the designed quinoilne-isatin conjugates **Q6a**–**h** and **Q8a**–**h** were synthesized and then biologically assessed for anti-tubercular actions towards drug-susceptible, MDR, and XDR strains. Superiorly, the *N*-benzyl-bearing compound **Q8b** possessed the best activities against the examined *M. tuberculosis* strains with MICs equal 0.06, 0.24, and 1.95 µg/mL, respectively.

## 1. Introduction

Tuberculosis (TB) remains one of the world’s most lethal infectious illnesses, killing an estimated 1.5 million people each year [1]. In 2018, the WHO reported a range of estimated incident cases between 9.0 and 11.1 million patients in addition to notifying 7.0 million new cases of TB [2]. The gap between the estimated incident cases and the new ones is obvious, showing the large global range of TB patients lacking the access to health care [3,4]. Furthermore, the situation worsened as a result of multiple aggravating factors, including difficulty in obtaining therapy, as well as the poor tolerability and efficacy of therapeutic regimens due to the administration of long-term drug combinations consisting of 1st-line medications (isoniazid, pyrazinamide, rifampicin, and ethambutol) alongside the more toxic, expensive, and less efficacious second-line agents (fluoroquinolones, aminoglycosides, and clofazimine). These drug protocols cause anti-tuberculosis drug-induced hepatotoxicity (ATDH), which causes significant morbidity and mortality while decreasing treatment effectiveness. Finally, incomplete treatment reduces effectiveness and eventually contributes to failure and relapse, which complicates disease resolution and results in serious TB drug resistance.

There is currently a substantial amount of information available regarding the spread of M. tuberculosis strains that are resistant to both first-line and second-line drugs for tuberculosis treatment. [5]. This resistance was studied and defined by the WHO into two types known as multi-drug-resistant tuberculosis (MDR-TB) that is considered to be resistant to rifampicin or isoniazid, and the extensively drug-resistant (XDR) tuberculosis that is able to resist also at least fluoroquinolone as well as either kanamycin or amikacin [6]. According to the World Health Organization (WHO), there were approximately 500,000 newly reported cases of rifampicin resistance (RR-TB) in 2018. Of these, 78% of the patients were MDR TB and 8.5% were XDR TB [2]. For patients suffering from XDR-TB, the efficiency of the standard treatments was quite limited. New hopes were provided through the approval of bedaquiline by the FDA. Rapidly, the failure of the clinically introduced drug by its first resistant *M. tuberculosis* isolate dramatically terminated the story [1]. As a result, the creation of novel anti-TB medications is urgently required. Novel drug candidates need to be able to shorten the therapeutic time period and simplify the actual treatment protocols, to enhance tolerance and to be effective towards resistant strains (MDR-TB and XDR-TB).

**Figure 1:**
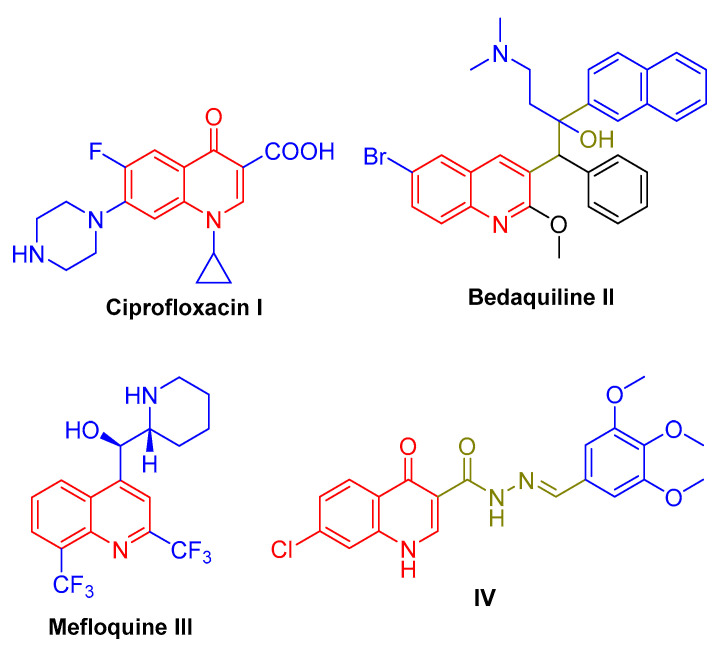
Structure for certain quinoline-based anti-tubercular drugs **I**–**III**, as well as the lead anti-tubercular compound **IV**.

Quinoline, a fused benzo[*b*]pyridine heterocycle, stands out as one of the most useful and versatile scaffolds endowed with different antibacterial activities. Interestingly, several quinoline-based anti-infectives are currently in clinical use as the antibacterial drug ciprofloxacin **I** (Figure 1), in addition to the recently identified anti-tubercular medications bedaquiline **II** and mefloquine **III** (Figure 1) [7,8,9,10]. Moreover, several research teams have reported a different series of small molecules tethered with the quinoline scaffold as promising anti-tubercular candidates [11,12,13,14,15,16,17,18,19]. In a recent study, novel small molecules bearing a quinoline-3-carbohydrazone moiety were synthesized and examined for the anti-tubercular impact on the drug-susceptible *M. tuberculosis* strain. The developed molecules exhibited moderate anti-tubercular activity and a disclosed MIC range of 6.24–99.84 µg/mL. Within this series, quinoline-derivative **IV** (Figure 1) disclosed the highest activity with a MIC value equal to 6.24 µg/mL [20].

Isatin is an endogenous motif that has been identified in many organisms. Isatin is found in numerous naturally occurring substances, including marine natural products, alkaloids, and fungal metabolites, in addition to being endogenously present in mammalian tissues and fluids, including those of humans and other mammals [21]. Isatin nucleus represents a versatile scaffold for chemical modifications, where its derivatives disclosed diverse pharmacological actions for the treatment of diverse diseases and disorders [21,22,23,24,25,26,27]. Isatin-tethered molecules such as nintedanib, sunitinib, semaxanib, and orantinib have either received clinical approval (nintedanib and sunitinib) or are currently the subject of clinical trials (semaxanib and orantinib) [28,29]. Such successful clinical applications, as well as their broad-spectrum pharmacological activities and the ease of the structural modifications, inspired and paved the way for the researchers to create a lot of isatin derivatives with significant structural diversity. In the last two decades, the isatin motif has attracted the researchers’ attention as a promising privileged skeleton that could be exploited for the discovery and development of tuberculosis medications. Accordingly, a large number of isatin-based series were developed and biologically explored for their potential anti-tubercular actions towards drug-susceptible strains as well as towards drug-resistant TB strains [30,31,32,33,34,35,36].

**Figure 2:**
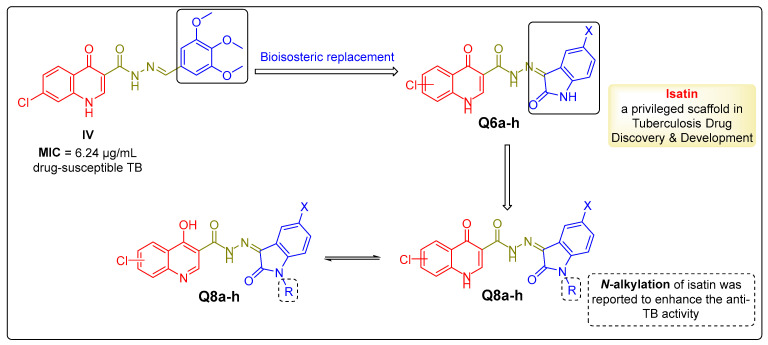
Design for the target quinoline-based molecules **Q6a**–**h** and **Q8a**–**h**.

In light of the aforementioned findings, and following up on our earlier efforts to identify promising anti-tubercular candidates [35,36,37,38,39,40,41], we decided to broaden our investigation by bioisosterically replacing the 3,4,5-trimethoxy benzylidene moiety in lead compound IV with the privileged isatin motif to afford the first series of targeted molecules **Q6a**–**h** (Figure 2). Furthermore, the isatin motif in series **Q6** was *N*-substituted with either a methyl or benzyl group to furnish the second series of target molecules **Q8a**–**h** (Figure 2). All of the designed quinoilne isatin conjugates **Q6a**–**h** and **Q8a**–**h** were synthesized and then biologically assessed for their anti-tubercular actions towards drug-susceptible MDR and XDR T.B. strains.

## 2. Results and Discussion

### 2.1. Synthestic Chemistry

**Scheme 1:**
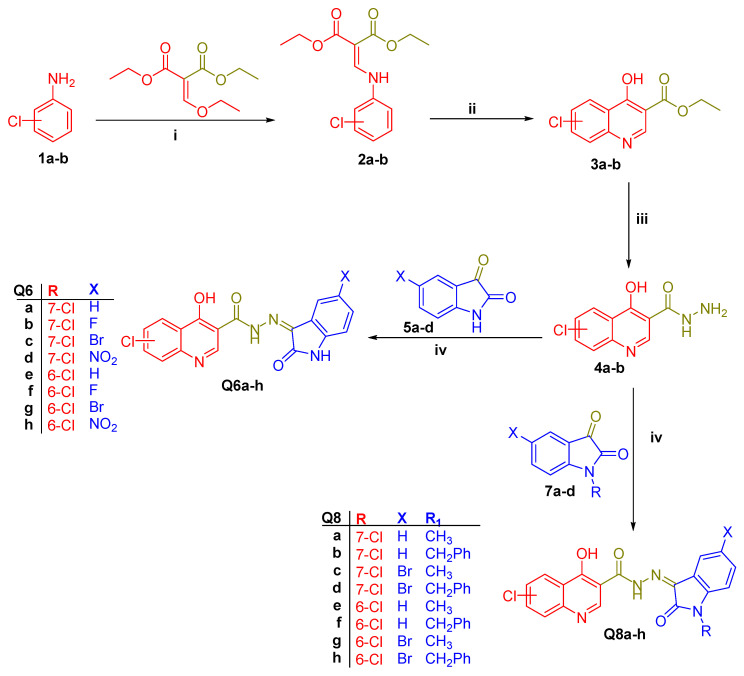
Synthetic route for target quinoline-isatin conjugates **Q6a**–**h** and **Q8a**–**h**; **i**: reflux 3 hours; **ii**: phenyl ether, reflux, 2 hours; **iii**: NH_2_NH_2_.H_2_O, reflux, 12 hours; and **iv**: glacial acetic acid, reflux, 4 hours.

The presented Scheme 1 illustrates the synthetic approach used to create the novel desired quinoline-isatin conjugates **Q6a**–**h** and **Q8a**–**h**. Ethyl anilinomethylene malonate **2a**–**b** was prepared through the condensation of 3-chloroaniline **1a** and 4-chloroaniline **1b** with diethyl ethoxymethylenemalonate. Then, the cyclization of **2a**–**b** in refluxing diphenyl ether produced the corresponding ethyl esters of 4-hydroxyquinoline-3-carboxylic acids **3a**–**b**, which then interacted with hydrazine hydrate (NH_2_NH_2_.H_2_O, 99%) in refluxing ethanol to produce the acid hydrazides **4a**–**b**. Finally, the target quinoline-isatin conjugates **Q6a**–**h** and **Q8a**–**h** were obtained by the condensation reaction of hydrazides **4a**–**b** with diverse 5-substituted isatins (**5a**–**d**) or *N*-substituted isatins (**7a**–**d**), respectively, in glacial acetic acid under refluxing temperature.

The IR spectra for quinoline-isatin conjugates **Q6a**–**h** and **Q8a**–**h** revealed absorption bands for the carbonyl functionality within the region 1629–1715 cm^−1^, beside the absorption bands that assigned to the amino (NH) functionalities in the 3249–3413 cm^−1^ region. The ^1^H NMR spectra for conjugates **Q6a**–**h** and **Q8a**–**h** exhibited two D_2_O-exchangeable singlets corresponding to the –CONH proton of Z and E isomers within the region *δ* 10.55–11.74 *ppm*, whereas another two D2O exchangeable singlet signals at *δ* 13.71–15.19 *ppm* were assigned for the Z and E isomers of OH quinoline. In addition, the NH protons of the isatin motif for derivatives **Q6a**–**h** were identified around *δ* 8.88–9.39 *ppm* as a singlet signals. The ^13^C NMR spectrum for conjugate **Q6b** disclosed characteristic signals resonating around *δ* 161.51–176.14 *ppm* that were attributable to the *Z* and *E* isomers of carbonyl groups carbons, while compounds **Q6f** and **Q6g** showed one isomer only, and the carbons of carbonyl (C=O) functionality appeared in the region *δ* 161.54–175.46 *ppm*.

### 2.2. Biological Evaluation

#### 2.2.1. Anti-Tubercular Effect for Quinolines **Q6a**–**h** and **Q8a**–**h**

The anti-tubercular activities for quinoline derivatives **Q6a**–**h** and **Q8a**–**h** towards RCMB 010126 *M. tuberculosis* were examined exploiting the microplate Alamar blue (MABA) protocol [42]. The used reference anti-tubercular drug was isoniazid. Table 1 summarises the anti-mycobacterial results, which are expressed as minimum inhibitory concentrations (MICs).

The outcomes demonstrated that the majority of the quinolines reported here had potent to moderate anti-*M. tuberculosis* activity, with the MICs range between 0.06 and 7.81 g/mL, with an exception for quinolines **Q6c** and **Q6d** (MIC = 31.25 and 15.63 µg/mL, respectively). Isatin-tethered derivative **Q8b** outperformed the lead compound **IV** in terms of growth inhibition towards *M. tuberculosis*, displaying a MIC of 0.06 g/mL, representing a 100-fold increase in activity (MIC for **IV** = 6.24 µg/mL). Moreover, compound **Q8h** also has potent anti-mycobacterial action with a MIC value of 0.12 µg/mL that equals the MIC for the standard isoniazide drug. In addition, quinolines **Q6a**, **Q6b**, **Q8a,** and **Q8f** exhibited potent anti-tubercular activity with MIC values in the range of 0.24–0.98 µg/mL, whereas quinolines **Q6e**–**Q6h**, **Q8c**, **Q8e**, and **Q8g** displayed moderate action with a range for the MIC values batween 1.95 and 7.81 µg/mL.

Studying the structural activity relationships of these quinoline-based hybrids revealed that their anti-tubercular actions have been influenced by two essential factors: the incorporation of a substituent at C-5 and N-1 of the oxindole motif, and the position of the chloro substituent (C-7 vs. C-6) at the quinoline moiety. With regard to the effect of the C-5 substitution within the oxindole motif within series **Q6**, it was found that grafting a 5-fluoro substituent resulted in an improved (for compound **Q6b**; MIC = 0.24 µg/mL) or maintained (for compound **Q6f**; MIC = 3.9 µg/mL) antitubercular impact in comparison to their unsubstituted analogues **Q6a** and **Q6e** (MICs = 0.48 and 3.9 µg/mL, respectively). The incorporation of 5-bromo (compounds **Q6c** and **Q6g**; MICs = 31.25 µg/mL and 7.81 µg/mL, respectively) or 5-nitro substituents (compounds **Q6d** and **Q6h**; MICs = 15.63 and 7.81 µg/mL, respectively), on the other hand, has resulted in a decreased anti-tubercular action in comparison to the unsubstituted analogues **Q6a** and **Q6e**.

Furthermore, upon investigation of the impact of the decoration of the oxindole motif with a 5-bromo substituent within series **8**, we found that such substitution has led to a decreased activity for 7-chloroquinoline-bearing derivatives **Q8c** and **Q8d** (MICs = 0.98 and 15.63 µg/mL, respectively) with regard to their unsubstituted counterparts **Q8a** and **Q8b** (MICs = 0.24 and 0.06 µg/mL, respectively), whereas this substitution maintained or enhanced the anti-tubercular activity for the 6-chloroquinoline-bearing derivatives **Q8g** and **Q8h** (MICs = 1.95 and 0.12 µg/mL, respectively) in comparison to their unsubstituted analogues **Q8e** and **Q8f** (MICs = 1.95 and 0.98 µg/mL, respectively).

Thereafter, we explored the obtained results to assess the effect of the *N*-methylation and *N*-benzylation of the oxindole motif on the anti-tubercular activity of series **8**. The results revealed that *N*-methylation enhanced the activity of both 7-chloroquinoline-bearing derivatives **Q8a** and **Q8c** (MICs = 0.24 and 0.98 µg/mL, respectively) and 6-chloroquinoline-bearing derivatives **Q8e** and **Q8g** (MICs = 1.95 µg/mL and 1.95 µg/mL, respectively). Similarly, the *N*-benzylation of the oxindole motif improved the activity of both the 7-chloroquinoline-bearing molecules **Q8b** and **Q8d** (MICs = 0.06 and 15.63 µg/mL, respectively) as well as the 6-chloroquinoline-bearing derivatives **Q8f** and **Q8h** (MICs = 0.98 µg/mL and 0.12 µg/mL, respectively). It is worth mentioning that the *N*-benzylated derivatives **Q8b**, **Q8f,** and **Q8h** (MICs = 0.06 µg/mL, 0.98 µg/mL, and 0.12 µg/mL) displayed better anti-tubercular activity than their corresponding *N*-methylated counterparts **Q8a**, **Q8e,** and **Q8g** (MICs = 0.24, 1.95, and 1.95 µg/mL).

Finally, upon comparing the anti-tubercular effect of 7-chloroquinoline-bearing derivatives **Q6a**–**Q6d** against their 7-chloroquinoline-bearing counterparts **Q6e**–**Q6h**, it was found that the introduction of the chloro substituent at C-7 is more beneficial for the activity of hybrids containing the un- and 5-F substituted oxindole motif (compounds **Q6a** and **Q6b**; MIC = 0.24 and 0.48 µg/mL, respectively), whereas grafting the chloro substituent at C-6 was found to be more advantageous than C-7 for hybrids endowed with the larger 5-Br and 5-NO_2_ substituents (compounds **Q6g** and **Q6h**; MIC = 7.81 µg/mL for both).

#### 2.2.2. Anti-Tubercular Activities toward MDR and XDR T.B 

The activity for the target quinolines **Q6a**–**h** and **Q8a**–**h** was assessed against the MDR strain (FJ05120) and the XDR strain (FJ05195). In Table 2, the assay outcomes are shown as minimum inhibitory concentration (MIC) values.

Compounds **Q8b** and **Q8h** showed superior activities against both MDR and XDR *M. tuberculosis* strains with MIC values equal to (0.24 µg/mL and 0.98 µg/mL) for MDR and (1.95 µg/mL, 3.9 µg/mL) for XDR, respectively. Additionally, quinolines **Q6a**, **Q6b**, **Q8a**, **Q8c**, **Q8e,** and **Q8f** exerted good activity against the MDR strain, with MIC values of 3.9, 3.9, 7.81, 3.9, 7.81, and 7.81 µg/mL, respectively (Table 2), whereas compounds **Q6a**, **Q6b**, **Q8c,** and **Q8f** displayed moderate anti-tubercular activity against the XDR strain, with a MIC range from 7.81 to 15.63 µg/mL.

#### 2.2.3. Cytotoxicity against Non-Tumorous Human Cells

In this study, T. Mosmann’s MTT colorimetric test was used to evaluate the cytotoxic potential of isatin-tethered quinoline derivatives **Q8b** and **Q8h** towards the human non-tumorigenic WI-38 lung fibroblast cell line [43] (Table 3). Notably, both examined quinolines **Q8b** and **Q8h** had no cytotoxic effect on WI-38 cells, with IC_50_ values of 36.6 ± 0.9 and 42.8 ± 1.12 μM, respectively.

## 3. Conclusions

Briefly, two sets of chemically synthesized isatin-tethered quinolines **Q6a**–**h** and **Q8a**–**h** were assessed for their anti-tubercular activities towards a drug-susceptible strain, as well as towards the MDR and XDR strains. The design of these molecules is based on the bioisosteric replacement for the 3,4,5-trimethoxy-benzylidene motif in the lead compound **IV** with the privileged isatin motif to furnish series **Q6**. Then, the isatin motif was *N*-substituted with either a methyl or benzyl group to furnish the second series **Q8**. The results demonstrated that the majority of the quinolines that were evaluated had powerful to moderate potency against drug-susceptible *M. tuberculosis*, with MICs ranging from 0.06 to 7.81 g/mL. Among the investigated molecules, compound **Q8b** is the most potent counterpart with MIC = 0.06 µg/mL; it represents about 100-fold enhanced activity compared to the lead compound **IV** (MIC = 6.24 µg/mL). Furthermore, the assessment of the activities against the MDR and XDR strains revealed that quinolines **Q8b** and **Q8h** showed superior activities against both MDR and XDR strains with MIC values equal to 0.24 µg/mL and 0.98 µg/mL for MDR and 1.95 µg/mL and 3.9 µg/mL for XDR, respectively. In the cytotoxicity MTT assay, quinolines **Q8b** and **Q8h** disclosed non-significant activity (IC_50_ = 36.6 ± 0.96 µM and 42.8 ± 1.12 µM, respectively) toward non-tumorigenic human WI-38 lung fibroblast cells.

## 4. Experimental

### 4.1. Chemistry

#### 4.1.1. General

Melting points (m.p.) were measured by a Stuart melting point apparatus and were uncorrected. The NMR spectra were recorded utilizing a Bruker 400 MHz FT-NMR spectrometer (Bruker, Billerica, MA, USA). Both ^1^H and ^13^C spectra were run at 400 MHz and 101 MHz, respectively, in DMSO-*d*_6_. Moreover, a Bruker FT-IR spectrophotometer was used to record the IR spectra.

#### 4.1.2. Preparation of 4-Hydroxyquinoline-3-Carboxylic Acid Ethyl Esters **3a**–**b**

A mixture of 3/4-chloroanilines **1a**–**b** (1.9 g, 15 mmol) and diethyl ethoxymethylenemalonate (4.3 g, 20 mmol) was heated at 130 °C for three hours, where the produced ethyl alcohol was evaporated off. The crude mixture was filtered by a sintered funnel, washed with petroleum ether, and dried. The malonates **2a**–**b** (3.6 g, 12 mol) were refluxed in diphenylether (40 mL) for 2 h. Upon cooling, the resultant solid was filtered off, dried, and recrystallized from acetonitrile to yield the corresponding hydroxyquinoline ethyl esters **3a**–**b** [44].

#### 4.1.3. Preparation of Quinoline-3-Carbohydrazide Derivatives **4a**–**b**

After adding hydrazine hydrate 99% (1 mL, 20 mmol) to a solution of quinoline ethyl esters **3a**–**b** (1 g, 4 mmol) in absolute ethanol (12 mL), the mixture was refluxed for 12 h. Before the mixture was added to the crushed ice, the excess solvent was removed. The solid that had precipitated was filtered through, washed with water, and then recrystallized from a mixture of DMF and water and dried to produce the corresponding hydroxyquinoline-3-carbohydrazides **4a**–**b** [44].

#### 4.1.4. Preparation of Target Quinoline-Isatin Conjugates **Q6a**–**h** and **Q8a**–**h**

The appropriate isatin derivative **5a**–**d** or **7a**–**b** (1.2 mmol) was added to a solution of quinoline-3-carbohydrazide derivatives **4a**–**b** (0.35 g, 1.5 mmol) in acetic acid (15 mL). This mixture was reflux-heated for 4 h. The target isatin-tethered quinolines **Q6a**–**h** and **Q8a**–**h** were obtained in 72–87% yield by recrystallizing the precipitate from DMF/ethanol after it had been filtered off while still hot and washed with hot ethanol.

### 4.2. Biological Evaluation

#### 4.2.1. Anti-Mycobacterial Activity

The MICs for the target isatin-tethered quinolines **Q6a**–**h** and **Q8a**–**h** against drug-susceptible, MDR, and XDR *M. tuberculosis* strains have been evaluated by the microplate alamar blue (MABA) assay, as described previously [36,45]. The detailed methodology was provided in the Supplementary Materials.

#### 4.2.2. MTT Colorimetric Assay

The cytotoxic activity for isatin-tethered quinolines **Q8b** and **Q8h** against lung WI-38 cells was assessed using T. Mosmann’s MTT colorimetric assay [43,46]. The detailed methodology was provided in the Supplementary Materials.

## Figures and Tables

**Table 1:** Anti-tubercular activity for the synthesized quinolines (**Q6a**–**h**) and (**Q8a**–**h**) against *M. tuberculosis* (MIC).

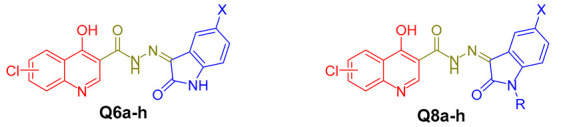				
Comp.	Cl	X	R	MIC
**Q6a**	7-Cl	H	–	0.48
**Q6b**	7-Cl	F	–	0.24
**Q6c**	7-Cl	Br	–	31.25
**Q6d**	7-Cl	NO_2_	–	15.63
**Q6e**	6-Cl	H	–	3.9
**Q6f**	6-Cl	F	–	3.9
**Q6g**	6-Cl	Br	–	7.81
**Q6h**	6-Cl	NO_2_	–	7.81
**Q8a**	7-Cl	H	-CH_3_	0.24
**Q8b**	7-Cl	H	-CH_2_-C_6_H_5_	0.06
**Q8c**	7-Cl	Br	-CH_3_	0.98
**Q8d**	7-Cl	Br	-CH_2_-C_6_H_5_	15.63
**Q8e**	6-Cl	H	-CH_3_	1.95
**Q8f**	6-Cl	H	-CH_2_-C_6_H_5_	0.98
**Q8g**	6-Cl	Br	-CH_3_	1.95
**Q8h**	6-Cl	Br	-CH_2_-C_6_H_5_	0.12
**Isoniazide**				0.12

**Table 2:** Anti-tubercular activities for the target quinolines (**Q6a**–**h**) and (**Q8a**–**h**) against MDR and XDR *M. tuberculosis*.

Comp.	MIC	
	MDR (FJ05120)	XDR (FJ05195)
**Q6a**	3.9	15.63
**Q6b**	3.9	7.81
**Q6c**	NA	N.A.
**Q6d**	62.5	N.A.
**Q6e**	15.63	125
**Q6f**	31.25	N.A.
**Q6g**	62.5	N.A.
**Q6h**	>125	N.A.
**Q8a**	7.81	31.25
**Q8b**	0.24	1.95
**Q8c**	3.9	15.63
**Q8d**	>125	N.A.
**Q8e**	7.81	31.25
**Q8f**	7.81	15.63
**Q8g**	15.63	>125
**Q8h**	0.98	3.9
**Isoniazid**	7.19	27.22

**Table 3:** Impact of quinolines **Q8b** and **Q8h** toward the human non-tumorigenic lung fibroblast WI-38 cell line.

Comp.	IC_50_ (µM)
	WI-38 Cells
**Q8b**	36.6 ± 0.96
**Q8h**	42.8 ± 1.12
**Staurosporine**	17.5 ± 0.46

## Data Availability

Not applicable.

## Supplementary Materials

The following supporting information can be downloaded at: https://www.mdpi.com/article/10.3390/molecules27248807/s1, Chapter S1. Characterisation details (NMR, IR and elemental analysis) for the target quinolines (**6a**–**h** and **8a**–**h**). Chapter S2. Microplate Alamar Blue Anti-Tubercular Assay. Chapter S3. MTT Cytotoxicity Assay. Figures S1–S19: The ^1^H, ^13^C NMR spectra for conjugates **Q6a**–**h** and **Q8a**–**h**. Figures S20–S23: The IR spectra for quinoline-isatin conjugates **6a,6d,6g,8h**.

## Sample Availability

Samples of the compounds are not available from the authors.

## References

[1] Coulibaly S., Cimino M., Ouattara M., Lecoutey C., Buchieri M.V., Alonso-Rodriguez N., Briffotaux J., Mornico D., Gicquel B., Rochais C. (2020). Phenanthrolinic analogs of quinolones show antibacterial activity against *M. tuberculosis*. Eur. J. Med. Chem..

[2] (2018). World Health Organization Global Tuberculosis Report. https://apps.who.int/iris/bitstream/handle/10665/274453/9789241565646-eng.pdf?sequence¼1&isAllowed¼y.

[3] (2019). World Health Organization Global Tuberculosis Report. https://apps.who.int/iris/bitstream/handle/10665/329368/9789241565714-eng.pdf?ua¼1.

[4] Ribeiro R.C., de Marins D.B., Di Leo I., Gomes L.D.S., de Moraes M.G., Abbadi B.L., Villela A.D., da Silva W.F., da Silva L.C.R., Machado P. (2020). Anti-tubercular profile of new selenium-menadione conjugates against Mycobacterium tuberculosis H37Rv (ATCC 27294) strain and multidrug-resistant clinical isolates. Eur. J. Med. Chem..

[5] Ghiano D.G., Recio-Balsells A., Bortolotti A., Defelipe L.A., Turjanski A., Morbidoni H.R., Labadie G.R. (2020). New one-pot synthesis of anti-tuberculosis compounds inspired on isoniazid. Eur. J. Med. Chem..

[6] Verma S.K., Verma R., Verma S., Vaishnav Y., Tiwari S., Rakesh K. (2020). Anti-tuberculosis activity and its structure-activity relationship (SAR) studies of oxadiazole derivatives: A key review. Eur. J. Med. Chem..

[7] Matviiuk T., Madacki J., Mori G., Orena B.S., Menendez C., Kysil A., André-Barrès C., Rodriguez F., Korduláková J., Mallet-Ladeira S. (2016). Pyrrolidinone and pyrrolidine derivatives: Evaluation as inhibitors of InhA and Mycobacterium tuberculosis. Eur. J. Med. Chem..

[8] Jayaprakash S., Iso Y., Wan B., Franzblau S., Kozikowski A.P. (2006). Design, Synthesis, and SAR Studies of Mefloquine-Based Ligands as Potential Antituberculosis Agents. Chemmedchem.

[9] Keri R.S., Patil S.A. (2014). Quinoline: A promising antitubercular target. Biomed. Pharmacother..

[10] Eswaran S., Adhikari A.V., Pal N.K., Chowdhury I.H. (2010). Design and synthesis of some new quinoline-3-carbohydrazone derivatives as potential antimycobacterial agents. Bioorganic Med. Chem. Lett..

[11] Briguglio I., Piras S., Corona P., Pirisi M., Jabes D., Carta A. (2012). SAR and Anti-Mycobacterial Activity of Quinolones and Triazoloquinolones: An Update. Anti-Infect. Agents.

[12] Singh S., Kaur G., Mangla V., Gupta M.K. (2015). Quinoline and quinolones: Promising scaffolds for future antimycobacterial agents. J. Enzym. Inhib. Med. Chem..

[13] Thomas K., Adhikari A.V., Telkar S., Chowdhury I.H., Mahmood R., Pal N.K., Row G., Sumesh E. (2011). Design, synthesis and docking studies of new quinoline-3-carbohydrazide derivatives as antitubercular agents. Eur. J. Med. Chem..

[14] Liu K.-L., Teng F., Xiong L., Li X., Gao C., Yu L.-T. (2021). Discovery of quinolone derivatives as antimycobacterial agents. RSC Adv..

[15] Imramovský A., Polanc S., Vinšová J., Kočevar M., Jampílek J., Rečková Z., Kaustová J. (2007). A new modification of anti-tubercular active molecules. Bioorg. Med. Chem..

[16] Nayyar A., Malde A., Coutinho E., Jain R. (2006). Synthesis, anti-tuberculosis activity, and 3D-QSAR study of ring-substituted-2/4-quinolinecarbaldehyde derivatives. Bioorg. Med. Chem..

[17] Venugopala K.N., Uppar V., Chandrashekharappa S., Abdallah H.H., Pillay M., Deb P.K., Morsy M.A., Aldhubiab B.E., Attimarad M., Nair A.B. (2020). Cytotoxicity and antimycobacterial properties of pyrrolo[1, 2-a] quinoline derivatives: Molecular target identification and molecular docking studies. Antibiotics.

[18] Insuasty D., Vidal O., Bernal A., Marquez E., Guzman J., Insuasty B., Quiroga J., Svetaz L., Zacchino S., Puerto G. (2019). Antimicrobial Activity of Quinoline-Based Hydroxyimidazolium Hybrids. Antibiotics.

[19] Upadhayaya R.S., Vandavasi J.K., Kardile R.A., Lahore S.V., Dixit S.S., Deokar H.S., Shinde P.D., Sarmah M.P., Chattopadhyaya J. (2010). Novel quinoline and naphthalene derivatives as potent antimycobacterial agents. Eur. J. Med. Chem..

[20] Abdelrahman M.A., Salama I., Gomaa M.S., Elaasser M.M., Abdel-Aziz M.M., Soliman D.H. (2017). Design, synthesis and 2D QSAR study of novel pyridine and quinolone hydrazone derivatives as potential antimicrobial and antitubercular agents. Eur. J. Med. Chem..

[21] Cheke R.S., Patil V.M., Firke S.D., Ambhore J.P., Ansari I.A., Patel H.M., Shinde S.D., Pasupuleti V.R., Hassan I., Adnan M. (2022). Therapeutic Outcomes of Isatin and Its Derivatives against Multiple Diseases: Recent Developments in Drug Discovery. Pharmaceuticals.

[22] Brandão P., Marques C., Burke A.J., Pineiro M. (2020). The application of isatin-based multicomponent-reactions in the quest for new bioactive and druglike molecules. Eur. J. Med. Chem..

[23] Chowdhary S., Shalini, Arora A., Kumar V. (2022). A Mini Review on Isatin, an Anticancer Scaffold with Potential Activities against Neglected Tropical Diseases (NTDs). Pharmaceuticals.

[24] Varun, Sonam, Kakkar R. (2019). Isatin and its derivatives: A survey of recent syntheses, reactions, and applications. MedChemComm.

[25] Cheke R.S., Firke S.D., Patil R.R., Bari S.B. (2018). Isatin: New hope against convulsion. Cent. Nerv. Syst. Agents Med. Chem. (Former. Curr. Med. Chem.-Cent. Nerv. Syst. Agents).

[26] Abdel-Aziz H., Ghabbour H.A., Eldehna W.M., Qabeel M.M., Fun H.K. (2014). Synthesis, crystal structure, and biological activity of cis/trans amide rotomers of (Z)-N′-(2-Oxoindolin-3-ylidene) formohydrazide. J. Chem..

[27] Taghour M.S., Elkady H., Eldehna W.M., El-Deeb N.M., Kenawy A.M., Elkaeed E.B., Alsfouk A.A., Alesawy M.S., Metwaly A.M., Eissa I.H. (2022). Design and synthesis of thiazolidine-2,4-diones hybrids with 1,2-dihydroquinolones and 2-oxindoles as potential VEGFR-2 inhibitors: In-vitro anticancer evaluation and in-silico studies. J. Enzym. Inhib. Med. Chem..

[28] Eldehna W.M., Al-Wabli R.I., Almutairi M.S., Keeton A.B., Piazza G.A., Abdel-Aziz H.A., Attia M.I. (2018). Synthesis and biological evaluation of certain hydrazonoindolin-2-one derivatives as new potent anti-proliferative agents. J. Enzym. Inhib. Med. Chem..

[29] Eldehna W.M., Fares M., Ibrahim H.S., Alsherbiny M.A., Aly M.H., Ghabbour H.A., Abdel-Aziz H.A. (2016). Synthesis and cytotoxic activity of biphenylurea derivatives containing indolin-2-one moieties. Molecules.

[30] Xu Z., Zhang S., Gao C., Fan J., Zhao F., Lv Z.-S., Feng L.-S. (2016). Isatin hybrids and their anti-tuberculosis activity. Chin. Chem. Lett..

[31] Jiang D., Wang G.-Q., Liu X., Zhang Z., Feng L.-S., Liu M.-L. (2018). Isatin Derivatives with Potential Antitubercular Activities. J. Heterocycl. Chem..

[32] Karalı N., Gürsoy A., Kandemirli F., Shvets N., Kaynak F.B., Özbey S., Kovalishyn V., Dimoglo A. (2007). Synthesis and structure–antituberculosis activity relationship of 1H-indole-2,3-dione derivatives. Bioorganic Med. Chem..

[33] Shahlaei M., Fassihi A., Nezami A. (2009). QSAR study of some 5-methyl/trifluoromethoxy-1H-indole-2,3-dione-3-thiosemicarbazone derivatives as anti-tubercular agents. Res. Pharm. Sci..

[34] Güzel Ö., Karalı N., Salman A. (2008). Synthesis and antituberculosis activity of 5-methyl/trifluoromethoxy-1H-indole-2,3-dione 3-thiosemicarbazone derivatives. Bioorganic Med. Chem..

[35] Eldehna W.M., El Hassab M.A., Abdelshafi N.A., Sayed F.A.-Z., Fares M., Al-Rashood S.T., Elsayed Z.M., Abdel-Aziz M.M., Elkaeed E.B., Elsabahy M. (2021). Development of potent nanosized isatin-isonicotinohydrazide hybrid for management of Mycobacterium tuberculosis. Int. J. Pharm..

[36] Elsayed Z.M., Eldehna W.M., Abdel-Aziz M.M., El Hassab M.A., Elkaeed E.B., Al-Warhi T., Abdel-Aziz H.A., Abou-Seri S.M., Mohammed E.R. (2021). Development of novel isatin–nicotinohydrazide hybrids with potent activity against susceptible/resistant Mycobacterium tuberculosis and bronchitis causing–bacteria. J. Enzym. Inhib. Med. Chem..

[37] Eldehna W.M., Fares M., Abdel-Aziz M.M., Abdel-Aziz H.A. (2015). Design, Synthesis and Antitubercular Activity of Certain Nicotinic Acid Hydrazides. Molecules.

[38] Abo-Ashour M.F., Eldehna W.M., George R.F., Abdel-Aziz M.M., Elaasser M.M., Abou-Seri S.M., Gawad N.M.A. (2018). Synthesis and biological evaluation of 2-aminothiazole-thiazolidinone conjugates as potential antitubercular agents. Futur. Med. Chem..

[39] Soliman D.H., Eldehna W.M., Ghabbour H.A., Kabil M.M., Abdel-Aziz M.M., Abdel-Aziz H.A. (2017). Novel 6-phenylnicotinohydrazide derivatives: Design, synthesis and biological evaluation as a novel class of antitubercular and antimicrobial agents. Biol. Pharm. Bull..

[40] Abdel-Aziz H.A., Eldehna W.M., Fares M., Al-Rashood S.T.A., Al-Rashood K.A., Abdel-Aziz M.M., Soliman D.H. (2015). Synthesis, biological evaluation and 2D-QSAR study of halophenyl bis-hydrazones as antimicrobial and antitubercular agents. Int. J. Mol. Sci..

[41] Abdel-Aziz H.A.K., Eldehna W.M., Fares M., Elsaman T., Abdel-Aziz M.M., Soliman D.H. (2015). Synthesis, in vitro and in silico studies of some novel 5-nitrofuran-2-yl hydrazones as antimicrobial and antitubercular agents. Biol. Pharm. Bull..

[42] Collins L., Franzblau S.G. (1997). Microplate alamar blue assay versus BACTEC 460 system for high-throughput screening of compounds against Mycobacterium tuberculosis and Mycobacterium avium. Antimicrob. Agents Chemother..

[43] Mosmann T. (1983). Rapid colorimetric assay for cellular growth and survival: Application to proliferation and cytotoxicity assays. J. Immunol. Methods.

[44] Srivatava N., Kumar A. (2013). Synthesis of substituted-4-oxo-1,4-dihydro-3-[1-oxo-2-hydrazino-3-{p-toluenesulfon}] quinoline Derivatives and their Biological Activity Against Bacterial Infections. Orient. J. Chem..

[45] Franzblau S.G., Witzig R.S., McLaughlin J.C., Torres P., Madico G., Hernandez A., Degnan M.T., Cook M.B., Quenzer V.K., Ferguson R.M. (1998). Rapid, Low-Technology MIC Determination with Clinical *Mycobacterium tuberculosis* Isolates by Using the Microplate Alamar Blue Assay. J. Clin. Microbiol..

[46] Hagras M.M.A., Deeb E., Elzahabi H.S.A., Elkaeed E.B., Mehany A.B.M., Eissa I.H. (2021). Discovery of new quinolines as potent colchicine binding site inhibitors: Design, synthesis, docking studies, and anti-proliferative evaluation. J. Enzym. Inhib. Med. Chem..

