# Supplementary material for: Development of Novel Isatin-Tethered Quinolines as Anti-Tubercular Agents against Multi and Extensively Drug-Resistant Mycobacterium tuberculosis
Source: Molecules. 2022 Dec 12;27(24):8807. doi: 10.3390/molecules27248807 (PMC9781264; doi:10.3390/molecules27248807)

Supporting Information

Development of novel isatin-tethered quinolines as anti-tubercular agents against multi and extensively drug-resistant Mycobacterium tuberculosis

Mohamed A. Abdelrahman*, Hadia Almahli, Tarfah Al-Warhi, Taghreed A. Majrashi, Marwa M. Abdel-Aziz, Wagdy M. Eldehna*, Mohamed A. Said

Tables of Contents

**1.**	Characterisation for the target quinolines (**6a-h** and **8a-h**)	2
**2.**	Microplate Alamar Blue Anti-Tubercular Assay	6
**3.**	MTT Cytotoxicity Assay	7
**4.**	NMR Spectra	8-26
**5**	IR Spectra	27-30

1. Characterisation details (NMR, IR and elemental analysis) for the target quinolines (6a-h and 8a-h)

7-Chloro-4-hydroxy-N'-(2-oxoindolin-3-ylidene)quinoline-3-carbohydrazide (**6a**)

Yellow crystal, E:Z ratio= 8:2 (yield 86%), m.p. above 300 °C; IR (KBr, ν cm^{-1}): 3451, 3184 (NH) and 1682, 1710 (C=O); ^{1}H NMR (DMSO-d_6) δ *ppm*: 6.91-7.20 (m, 2.5H, Ar-H), 7.33-7.52, (m, 2.5H, Ar-H), 7.70-7.77 (m, 1H, Ar-H), 8.25-8.48 (m, 2H, Ar-H), 8.89, 9.01 (2s, 1H, NH-Isatin, D_2O exchangeable), 10.80, 11.03 (2s, 1H, –CONH, D_2O exchangeable), 13.79, 14.97 (2s, 1H, OH- quinoline, D_2O exchangeable); Anal. calcd. for $C_{18}H_{11}ClN_4O_3$ (366.76): C, 58.95; H, 3.02; N, 15.28. Found C, 59.17; H, 3.05; N, 15.37.

7-Chloro-N'-(5-fluoro-2-oxoindolin-3-ylidene)-4-hydroxyquinoline-3-carbohydrazide (**6b**)
Yellow crystal, E:Z ratio= 4:6 (yield 73%), m.p. above 300 °C; IR (KBr, ν cm^{-1}): 3303, 3249 (NH) and 1711, 1629 (C=O); ^{1}H NMR (DMSO-d_6) δ *ppm*: 6.89-6.94 (m, 1H, Ar-H), 7.17-7.35 (m, 2H, Ar-H), 7.51 (d, 1H, J = 8.4 Hz, Ar-H), 7.75 (s, 1H, Ar-H), 8.25-8.32 (m, 2H, Ar-H), 8.90, 9.02 (2s, 1H, NH-Isatin, D_2O exchangeable), 10.83, 11.07 (2s, 1H, –CONH, D_2O exchangeable), 13.73, 15.00 (2s, 1H, OH- quinoline, D_2O exchangeable); ^{13}C NMR (DMSO-d_6) δ *ppm*: 108.05, 108.30, 110.44, 110.74, 112.24, 116.10, 118.14, 118.91, 119.13, 122.37, 125.21, 125.40, 126.16, 126.54, 128.36, 136.85, 138.01, 138.36, 139.21, 140.34, 141.10, 146.71, 146.89, 157.32, 157.45, 159.81, 161.51, 163.09, 165.49, 175.31, 176.14; Anal. calcd. for $C_{18}H_{10}ClFN_4O_3$ (384.75): C, 56.19; H, 2.62; N, 14.56. Found C, 56.33; H, 2.61; N, 14.61.

N'-(5-Bromo-2-oxoindolin-3-ylidene)-7-chloro-4-hydroxyquinoline-3-carbohydrazide (**6c**)
Yellow crystal, E:Z ratio= 5.5:4.5 (yield 79%), m.p. above 300 °C; IR (KBr, ν cm^{-1}): 3312, 3250 (NH) and 1715, 1662 (C=O); ^{1}H NMR (DMSO-d_6) δ *ppm*: 6.89-6.94 (m, 1H, Ar-H), 7.52-7.58 (m, 1H, Ar-H), 7.63-7.66 (m, 2H, Ar-H), 7.80-7.85 (m, 1H, Ar-H), 8.29-8.32 (m, 1.5H, Ar-H), 8.71 (s, 0.5H, Ar-H), 8.96, 9.09 (2s, 1H, NH-Isatin, D_2O exchangeable), 10.98, 11.21 (2s, 1H, –CONH, D_2O exchangeable), 13.87, 15.04 (2s, 1H, OH- quinoline, D_2O exchangeable); Anal. calcd. for $C_{18}H_{10}BrClN_4O_3$ (445.65): C, 48.51; H, 2.26; N, 12.57. Found C, 58.63; H, 2.28; N, 12.52.

7-Chloro-4-hydroxy-N'-(5-nitro-2-oxoindolin-3-ylidene)quinoline-3-carbohydrazide (**6d**)

Yellow crystal, E:Z ratio= 5.7:4.3 (yield 83%), m.p. above 300 °C; IR (KBr, ν cm^{-1}): 3450, 3236 (NH) and 1701, 1682 (C=O); ^{1}H NMR (DMSO-d_6) *δ ppm*: 7.07-7.11 (m, 1H, Ar-H), 7.49-7.79 (m, 2H, Ar-H), 8.21-8.35 (m, 2H, Ar-H), 8.87-8.92 (m, 1H, Ar-H), 9.03, 9.39 (2s, 1H, NH-Isatin, D_2O exchangeable), 10.55-10.66 (m, 1H, Ar-H), 11.50, 11.73 (2s, 1H, –CONH, D_2O exchangeable), 14.1, 15.00 (2s, 1H, OH- quinoline, D_2O exchangeable); Anal. calcd. for $C_{18}H_{10}ClN_5O_5$ (411.75): C, 52.51; H, 2.45; N, 17.01. Found C, 52.69; H, 2.48; N, 16.93.

6-Chloro-4-hydroxy-N'-(2-oxoindolin-3-ylidene)quinoline-3-carbohydrazide (**6e**)
Yellow crystal, E:Z ratio= 7.7:2.3 (yield 85%), m.p. above 300 °C; IR (KBr, ν cm^{-1}): 3309, 3247 (NH) and 1713, 1634 (C=O); ^{1}H NMR (DMSO-d_6) *δ ppm*: 6.91, 6.95 (d, 1H, *J* = 8.0 Hz, Ar-H), 7.03, 7.21 (t, 1H, *J* = 8.0 Hz, *J* = 16.0 Hz, Ar-H), 7.34-7.58 (m, 2H, Ar-H), 7.75-7.85 (m, 2H, Ar-H), 8.20-8.46 (m, 2H, Ar-H), 8.88-9.00 (s, 1H, NH-Isatin, D_2O exchangeable), 10.80, 11.04 (2s, 1H, –CONH, D_2O exchangeable), 13.73, 14.97 (2s, 1H, OH- quinoline, D_2O exchangeable); Anal. calcd. for $C_{18}H_{11}ClN_4O_3$ (366.76): C, 58.95; H, 3.02; N, 15.28. Found C, 59.14; H, 3.05; N, 15.37.

6-Chloro-N'-(5-fluoro-2-oxoindolin-3-ylidene)-4-hydroxyquinoline-3-carbohydrazide (**6f**).
Yellow crystal, E:Z ratio= 3.6:6.4 (yield 71%), m.p. above 300 °C; IR (KBr, ν cm^{-1}): 3410, 3294 (NH) and 1706, 1636 (C=O); ^{1}H NMR (DMSO-d_6) *δ ppm*: 6.92 (s, 1H, Ar-H), 7.20-7.34 (m, 2H, Ar-H), 7.77-7.81 (m, 2H, Ar-H), 8.20-8.29 (m, 2H, Ar-H), 8.90, 9.03 (2s, 1H, NH-Isatin, D_2O exchangeable), 10.83, 11.09 (2s, 1H, –CONH, D_2O exchangeable), 13.71, 15.01 (2s 1H, OH- quinoline, D_2O exchangeable); ^{13}C NMR (DMSO-d_6) *δ ppm*: 108.31, 110.44, 112.23, 122.10, 122.37, 124.84, 125.06, 130.54, 133.55, 136.59, 138.20, 138.96, 140.47, 146.29, 161.54, 163.07; Anal. calcd. for $C_{18}H_{10}ClFN_4O_3$ (384.75): C, 56.19; H, 2.62; N, 14.56. Found C, 56.34; H, 2.59; N, 15.61.

N'-(5-Bromo-2-oxoindolin-3-ylidene)-7-chloro-4-hydroxyquinoline-3-carbohydrazide (**6g**).
Yellow crystal, E:Z ratio= 6:4 (yield 82%), m.p. above 300 °C; IR (KBr, ν cm^{-1}): 3449, 3242 (NH) and 1712, 1656 (C=O); ^{1}H NMR (DMSO-d_6) *δ ppm*: 6.89-6.94 (m, 1H, Ar-H), 7.52-7.58 (m, 1H, Ar-H), 7.63-7.66 (m, 2H, Ar-H), 7.80-7.85 (m, 1H, Ar-H), 8.29-8.32 (m, 1.5H, Ar-H), 8.71 (s, 0.5H, Ar-H), 8.96, 9.09 (2s, 1H, NH-Isatin, D_2O exchangeable), 10.98, 11.21 (2s, 1H, –CONH, D_2O exchangeable), 13.87, 15.04 (2s, 1H, OH- quinoline, D_2O exchangeable); ^{13}C NMR

(DMSO-d_6) δ *ppm*: 110.25, 114.15, 117.88, 121.27, 124.72, 124.83, 127.20, 127.42, 127.51, 130.88, 133.29, 137.34, 143.46, 146.63, 163.13, 175.46; Anal. calcd. for $C_{18}H_{10}BrClN_4O_3$ (445.65): C, 48.51; H, 2.26; N, 12.57. Found C, 48.63; H, 2.28; N, 12.52.

6-Chloro-4-hydroxy-N'-(5-nitro-2-oxoindolin-3-ylidene)quinoline-3-carbohydrazide (**6h**).
Yellow crystal, E:Z ratio= 5:5 (yield 85%), m.p. above 300 °C; IR (KBr, ν cm^{-1}): 3319, 3249 (NH, NH) and 1715, 1660 (C=O); ^{1}H NMR (DMSO-d_6) δ *ppm*: 7.12-7.17 (m, 0.5H, Ar-H), 7.76-7.93 (m, 3H, Ar-H), 8.19-8.41 (m, 2H, Ar-H), 8.79-8.83 (m, 1H, Ar-H), 8.98-9.52 (m, 0.5H, Ar-H), 10.54-10.60 (m, 1H, NH-Isatin, D_2O exchangeable), 11.55, 11.72 (2s, 1H, –CONH, D_2O exchangeable), 14.27, 15.10 (2s, 1H, OH- quinoline, D_2O exchangeable); Anal. calcd. for $C_{18}H_{10}ClN_5O_5$ (411.75): C, 52.51; H, 2.45; N, 17.01. Found C, 52.39; H, 2.46; N, 16.93.

7-Chloro-4-hydroxy-N'-(1-methyl-2-oxoindolin-3-ylidene)quinoline-3-carbohydrazide (**8a**)
Yellow crystal, E:Z ratio= 6:4 (yield 87%), m.p. above 300 °C; IR (KBr, ν cm^{-1}): 3316, 3245 (NH) and 1708, 1640 (C=O); ^{1}H NMR (DMSO-d_6) δ *ppm*: 3.37 (s, 3H, CH_3), 7.14-7.33 (m, 1.5H, Ar-H), 7.46-7.65 (m, 2H, Ar-H), 7.78-7.86 (m, 1H, Ar-H), 8.25-8.57 (m, 2H, Ar-H), 8.80-9.01 (m, 1.5H, Ar-H), 10.61, 11.74 (2s, 1H, –CONH, D_2O exchangeable), 14.01, 15.11 (2s, 1H, OH- quinoline, D_2O exchangeable); Anal. calcd. for $C_{19}H_{13}ClN_4O_3$ (380.78): C, 59.93; H, 3.44; N, 14.71. Found C, 60.08; H, 3.47; N, 14.65.

N'-(1-Benzyl-2-oxoindolin-3-ylidene)-7-chloro-4-hydroxyquinoline-3-carbohydrazide (**8b**)
Yellow crystal, E:Z ratio= 5:5 (yield 84%), m.p. above 300 °C; IR (KBr, ν cm^{-1}): 3310, 3228 (NH) and 1711, 1640 (C=O); ^{1}H NMR (DMSO-d_6) δ *ppm*: 5.02-5.05 (m, 2H, CH_2), 7.09-9.11 (m, 13H, Ar-H), 10.55, 11.73 (2s, 1H, –CONH, D_2O exchangeable), 14.02, 15.16 (2s, 1H, OH-quinoline, D_2O exchangeable); Anal. calcd. for $C_{25}H_{17}ClN_4O_3$ (456.88): C, 65.72; H, 3.75; N, 7.76. Found C, 65.85; H, 3.72; N, 7.67.

N'-(5-Bromo-1-methyl-2-oxoindolin-3-ylidene)-7-chloro-4-hydroxyquinoline-3-carbohydrazide (**8c**)
Yellow crystal, E:Z ratio= 5:5 (yield 81%), m.p. above 300 °C; IR (KBr, ν cm^{-1}): 3313, 3235 (NH) and 1708, 1649 (C=O); ^{1}H NMR (DMSO-d_6) δ *ppm*: 3.35 (s, 3H, CH_3), 6.89-6.95 (m, 1H, Ar-H), 7.52-7.58 (m, 1H, Ar-H), 7.63-7.66 (m, 1.5H, Ar-H), 7.80-7.85 (m, 1H, Ar-H), 8.27-8.39

(m, 1H, Ar-H), 8.71 (s, 0.5H, Ar-H), 8.96, 9.09 (2s, 1H, Ar-H), 10.98, 11.21 (2s, 1H, –CONH, D_2O exchangeable), 13.87, 15.04 (2s, 1H, OH- quinoline, D_2O exchangeable); Anal. calcd. for $C_{19}H_{12}BrClN_4O_3$ (459.68): C, 49.64; H, 2.63; N, 12.19. Found C, 49.75; H, 2.62; N, 12.25.

N'-(1-Benzyl-5-bromo-2-oxoindolin-3-ylidene)-7-chloro-4-hydroxyquinoline-3-carbohydrazide (**8d**)

Yellow crystal, E:Z ratio= 4:6 (yield 79%), m.p. above 300 °C; IR (KBr, ν cm^{-1}): 3319, 3248 (NH) and 1710, 1651 (C=O); ^1H NMR (DMSO-d_6) δ *ppm*: 5.02-5.04 (m, 2H, CH_2), 7.02 (t, 1H, J = 8.0 Hz, Ar-H), 7.27-7.41 (m, 4H, Ar-H), 7.54-7.85 (m, 4H, Ar-H), 8.27-8.38 (m, 1H, Ar-H), 8.80-9.11 (m, 2H, Ar-H), 10.54, 11.73 (s, 1H, –CONH, D_2O exchangeable), 14.13, 15.18 (s, 1H, OH- quinoline, D_2O exchangeable); Anal. calcd. for $C_{25}H_{16}BrClN_4O_3$ (535.78): C, 56.04; H, 3.01; N, 10.46. Found C, 56.14; H, 3.03; N, 10.57.

6-Chloro-4-hydroxy-N'-(1-methyl-2-oxoindolin-3-ylidene)quinoline-3-carbohydrazide (**8e**).

Yellow crystal, E:Z ratio= 5.5:4.5 (yield 85%), m.p. above 300 °C; IR (KBr, ν cm^{-1}): 3321, 3232 (NH) and 1703, 1648 (C=O); ^1H NMR (DMSO-d_6) δ *ppm*: 3.31 (s, 3H, CH_3), 7.14-7.36 (m, 1.5H, Ar-H), 7.46-7.64 (m, 1.5H, Ar-H), 7.76-7.92 (m, 2.5H, Ar-H), 8.25-8.56 (m, 1.5H, Ar-H), 8.79-9.08 (m, 1H, Ar-H), 10.60, 11.70 (2s, 1H, –CONH, D_2O exchangeable), 13.93, 15.09 (2s, 1H, OH- quinoline, D_2O exchangeable); Anal. calcd. for $C_{19}H_{13}ClN_4O_3$ (380.78): C, 59.93; H, 3.44; N, 14.71. Found C, 59.97; H, 3.45; N, 14.67.

N'-(1-Benzyl-2-oxoindolin-3-ylidene)-6-chloro-4-hydroxyquinoline-3-carbohydrazide (**8f**).

Yellow crystal, E:Z ratio= 6:4 (yield 82%), m.p. above 300 °C; IR (KBr, ν cm^{-1}): 3323, 3236 (NH) and 1707, 1643 (C=O); ^1H NMR (DMSO-d_6) δ *ppm*: 5.03-5.05 (m, 2H, CH_2), 7.09-9.07 (m, 13H, Ar-H), 10.52, 11.74 (2s, 1H, –CONH, D_2O exchangeable), 14.31, 15.19 (2s, 1H, OH- quinoline, D_2O exchangeable); Anal. calcd. for $C_{25}H_{17}ClN_4O_3$ (456.88): C, 65.72; H, 3.75; N, 7.76. Found C, 65.85; H, 3.62; N, 7.67.

N'-(5-Bromo-1-methyl-2-oxoindolin-3-ylidene)-6-chloro-4-hydroxyquinoline-3-carbohydrazide (**8g**).

Yellow crystal, E:Z ratio= 3:7 (yield 80%), m.p. above 300 °C; IR (KBr, ν cm^{-1}): 3319, 3247 (NH) and 1701, 1636 (C=O); ^1H NMR (DMSO-d_6) δ *ppm*: 3.24 (s, 3H, CH_3), 7.09-7.13 (m, 1H, Ar-H), 7.49-7.80 (m, 3H, Ar-H), 8.23-8.32 (m, 1H, Ar-H), 8.70-9.04 (m, 2H, Ar-H), 10.98,

11.21 (2s, 1H, –CONH, D_2O exchangeable), 13.87, 15.04 (2s, 1H, OH- quinoline, D_2O exchangeable); Anal. calcd. for $C_{19}H_{12}BrClN_4O_3$ (459.68): C, 49.64; H, 2.63; N, 12.19. Found C, 49.75; H, 2.61; N, 12.25.

N'-(1-Benzyl-5-bromo-2-oxoindolin-3-ylidene)-6-chloro-4-hydroxyquinoline-3-carbohydrazide (**8h**).

Yellow crystal, E:Z ratio= 2:8 (yield 81%), m.p. above 300 °C; IR (KBr, ν cm^{-1}): 3449, 3244 (NH) and 1709, 1683 (C=O); ^1H NMR (DMSO-d_6) δ *ppm*: 5.02-5.04 (m, 2H, CH_2), 7.03-7.05 (m, 1H, Ar-H), 7.31-7.42 (m, 5H, Ar-H), 7.56-7.58 (m, 1H, Ar-H), 7.71-7.87 (m, 3H, Ar-H), 8.19-8.24 (m, 1H, Ar-H), 8.80-9.09 (m, 1H, Ar-H), 10.54, 11.73 (2s, 1H, –CONH, D_2O exchangeable), 14.13, 15.18 (2s, 1H, OH- quinoline, D_2O exchangeable); Anal. calcd. for $C_{25}H_{16}BrClN_4O_3$ (535.78): C, 56.04; H, 3.01; N, 10.46. Found C, 55.88; H, 3.03; N, 10.53.

2. Microplate Alamar Blue Anti-Tubercular Assay

Microplate alamar blue assay (MABA) was used to determine the MICs for the prepared (**6a-h** and **8a-h**) against drug-susceptible strain (ATCC 25177), MDR (ATCC 35822; isoniazid, cycloserine, kanamycin, and rifampin-resistant), XDR (RCMB 2674; isoniazid, rifampicin, ethambutol, pyrazinamide, ethionamide, and moxifloxacin-resistant) M. tuberculosis strains. Isoniazid was used as a reference drug against sensitive strain. Preparation of the inoculum was done using fresh Lowenstein Jensen (LJ) medium re-suspended in 7H9-S medium (7H9 broth, 0.1% casitone, 0.5% glycerol, supplemented oleic acid, albumin, dextrose, and catalase (OADC), adjusted to a McFarland tube No. 1, and diluted 1:20; 100 µl was used as inoculum. The examined hybrids were dissolved in DMSO. Drug-free controls containing broth with DMSO were included in the experiment. The final concentration of DMSO in the test medium did not exceed 0.5% (v/v) of the total solution composition, which had no effect on the growth of *M. tuberculosis*. The 96 wells plates were treated with 100 µl of two-fold serial dilution of each compound. Final concentrations of the examined hybrids in wells were 1000- 0.006 µg/mL. Sterile deionized water (200 µl) was added to all outer-perimeter wells of sterile 96 well plates to decrease evaporation of the medium in the test wells during incubation. A growth control without antibiotic and a sterile control were also set on each plate. The plate was then covered, sealed in plastic bags and incubated at 37 °C in normal atmosphere. After 7 days of incubation, each well was supplied with 30 µl of the Alamar blue solution, and then the plate was re-

incubated overnight. Colour change from blue (oxidized state) to pink (reduced) highlighted the growth of bacteria. The MIC was expressed as the minimum concentration of compound which prohibited blue to pink colour change. MIC values were calculated in µg/mL.

3. MTT Cytotoxicity Assay

The examined lung fibroblast WI-38 cell have been obtained from American Type Culture Collection (ATCC). Cells lines were maintained as monolayers in Dulbecco's Modified Eagle's Medium (DMEM) supplemented with 10% FBS, 2 mM L-glutamine, 100 U/ml penicillin and 100µg/ml streptomycin sulfate. Cells were sub-cultured with trypsine /EDTA solution, counted with haemocytometer and plated onto 96-well plates (5000 cells/well) and left overnight to form a semi-confluent monolayer. Cell monolayers were treated in quadrates with vehicle (DMSO, 0.1% v/v), test samples or Adriamycin as positive control for an exposure time of 48 h. At the end of exposure, MTT solution in PBS (5 mg/ml) was then added to all well including no cell blank and left to incubate for 90 min. The formation of formazan crystals were visually confirmed using phase contract microscopy. DMSO (100 µl/well) was added to dissolve the formazan crystals with shaking for 10 min after which the absorbance was read at 590 nm against no cell blanks on a FLuo Star Optima microplate reader (BMG technologies, Germany). Cell proliferation was calculated comparing the OD values of the DMSO control wells and those of the samples represented as % proliferation to the control. Dose-response experiment was performed on samples producing > or =50% loss of cell proliferation using five serial 2-fold dilutions (50, 25, 12.5, 6.25 and 3.125 µM) of the sample. IC_{50} values (concentration of sample causing 50% loss of cell proliferation of the vehicle control) were calculated using non-linear regression curve fitting of the dose response plots on GraphPad Prism V.6.0 software.

Figure S1: The 1H NMR spectra for 6a

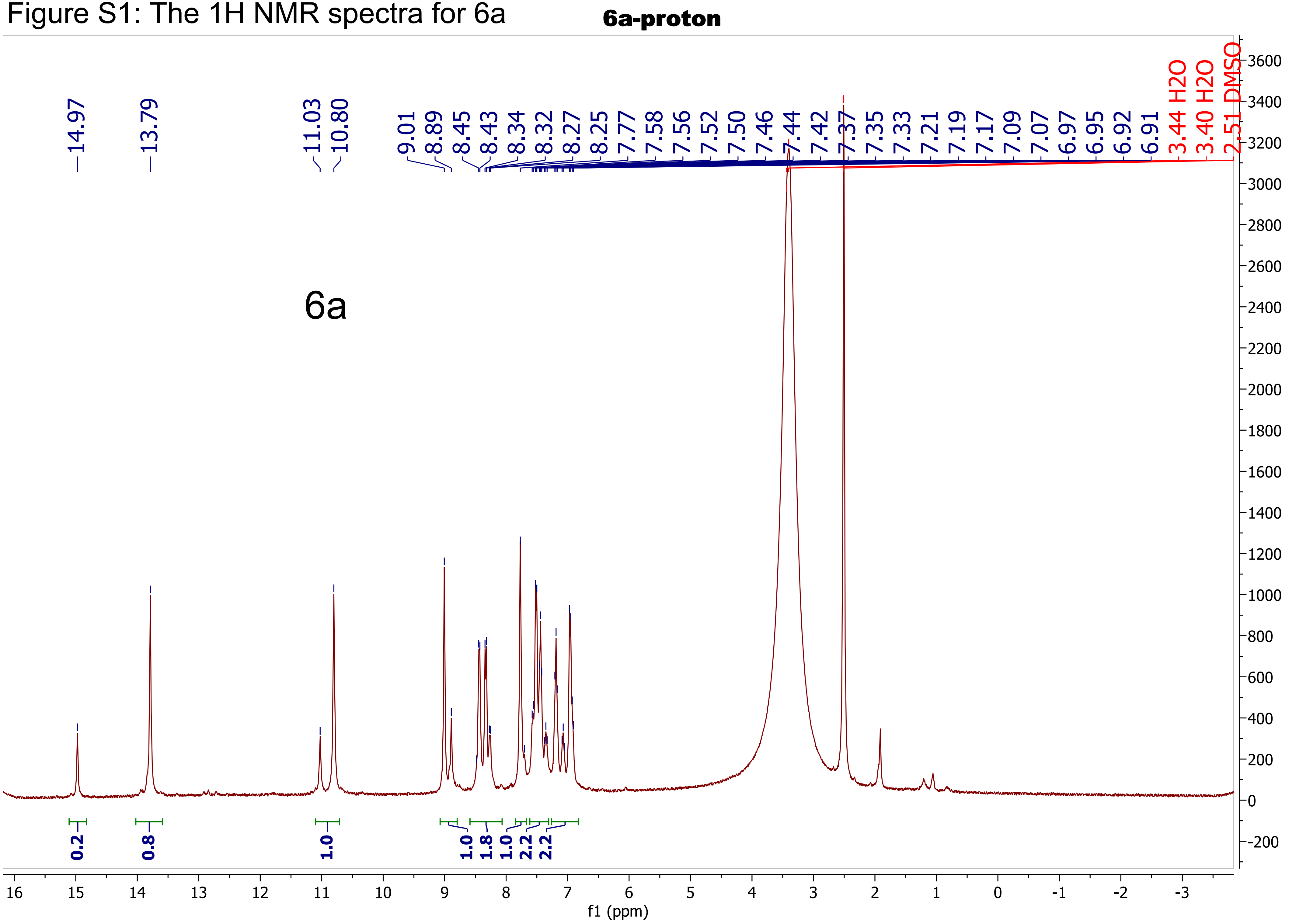

Figure S2: The 1H NMR spectra for 6b

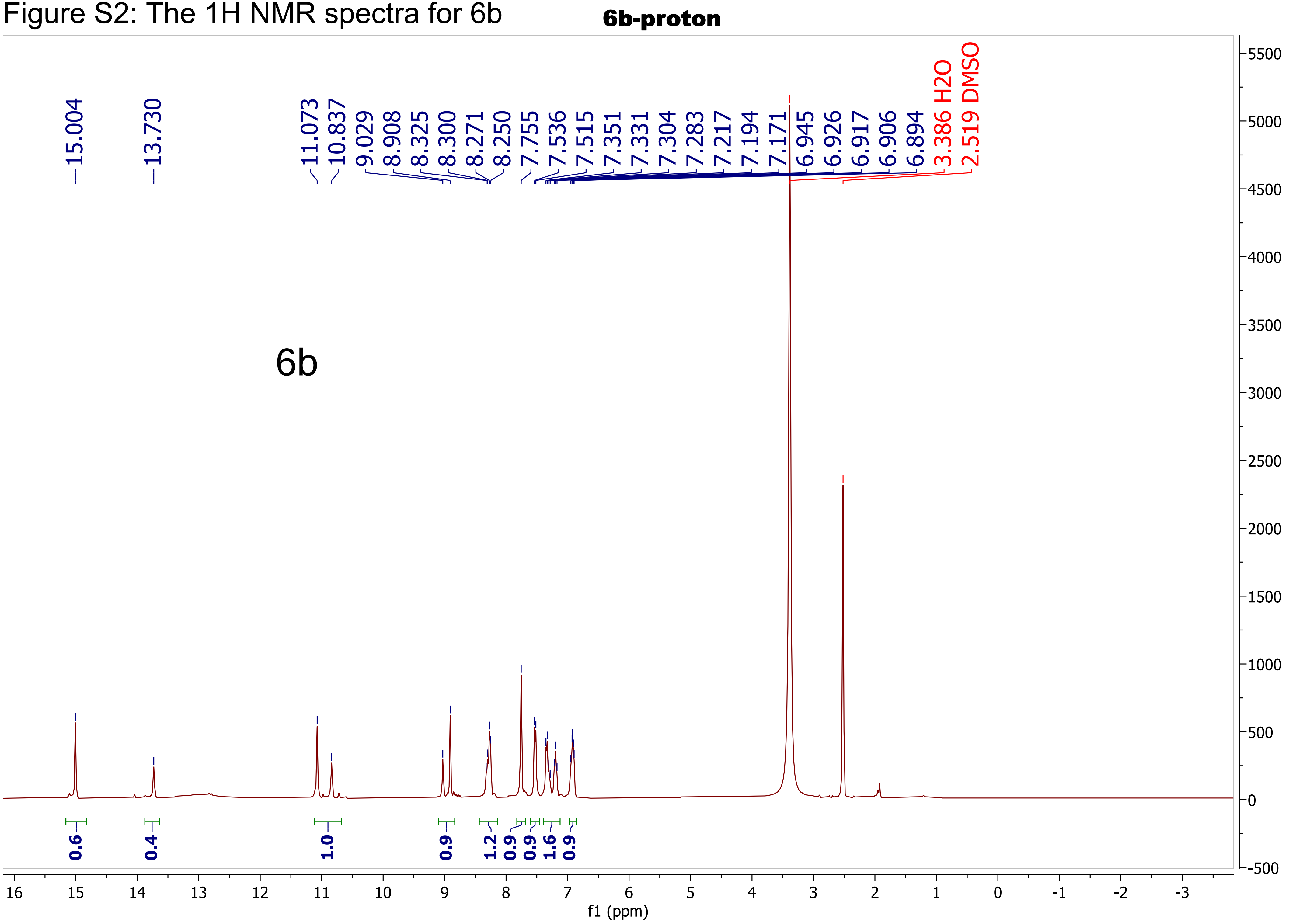

Figure S3: The 13C NMR spectra for 6b

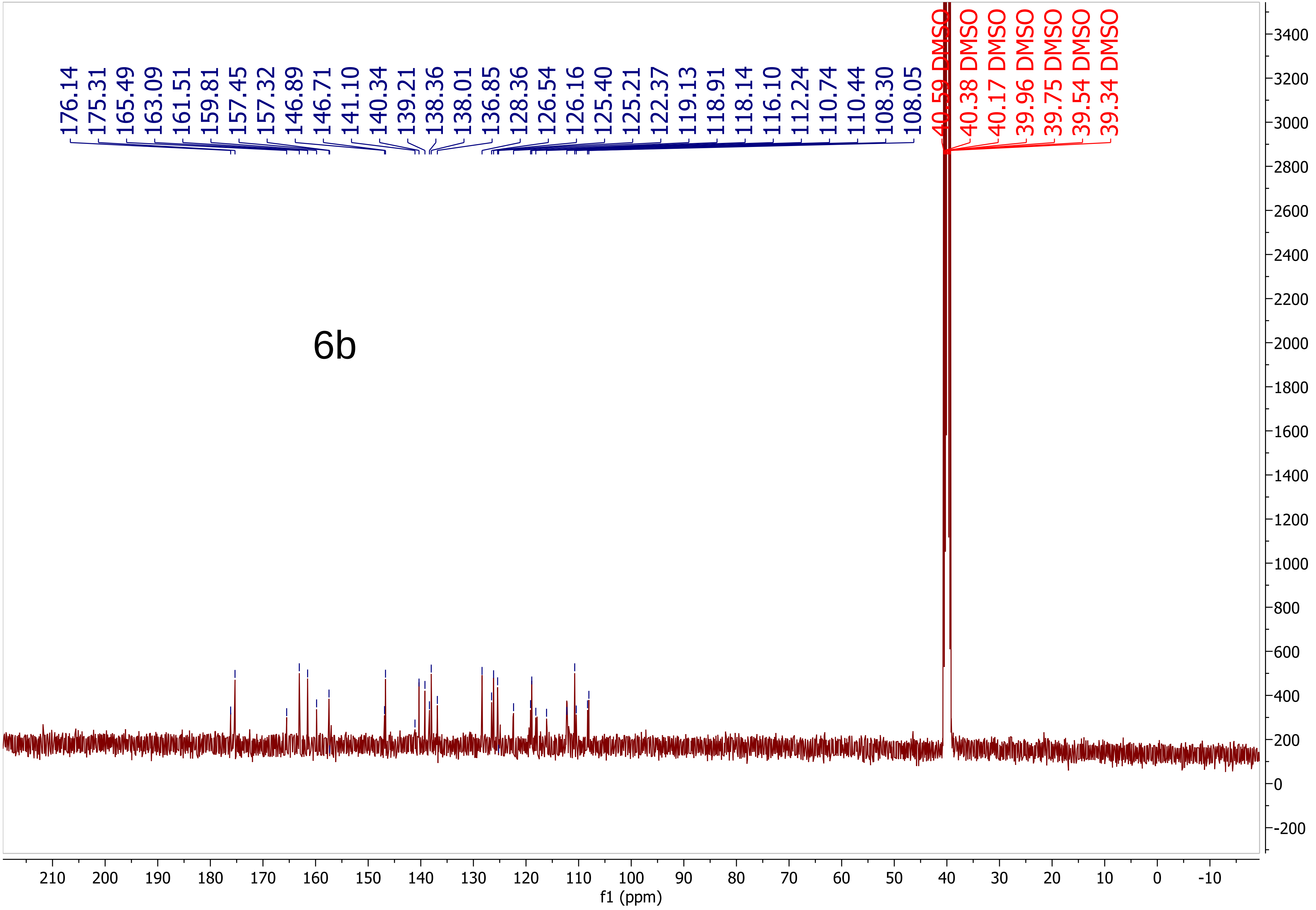

Figure S4: The 1H NMR spectra for 6c

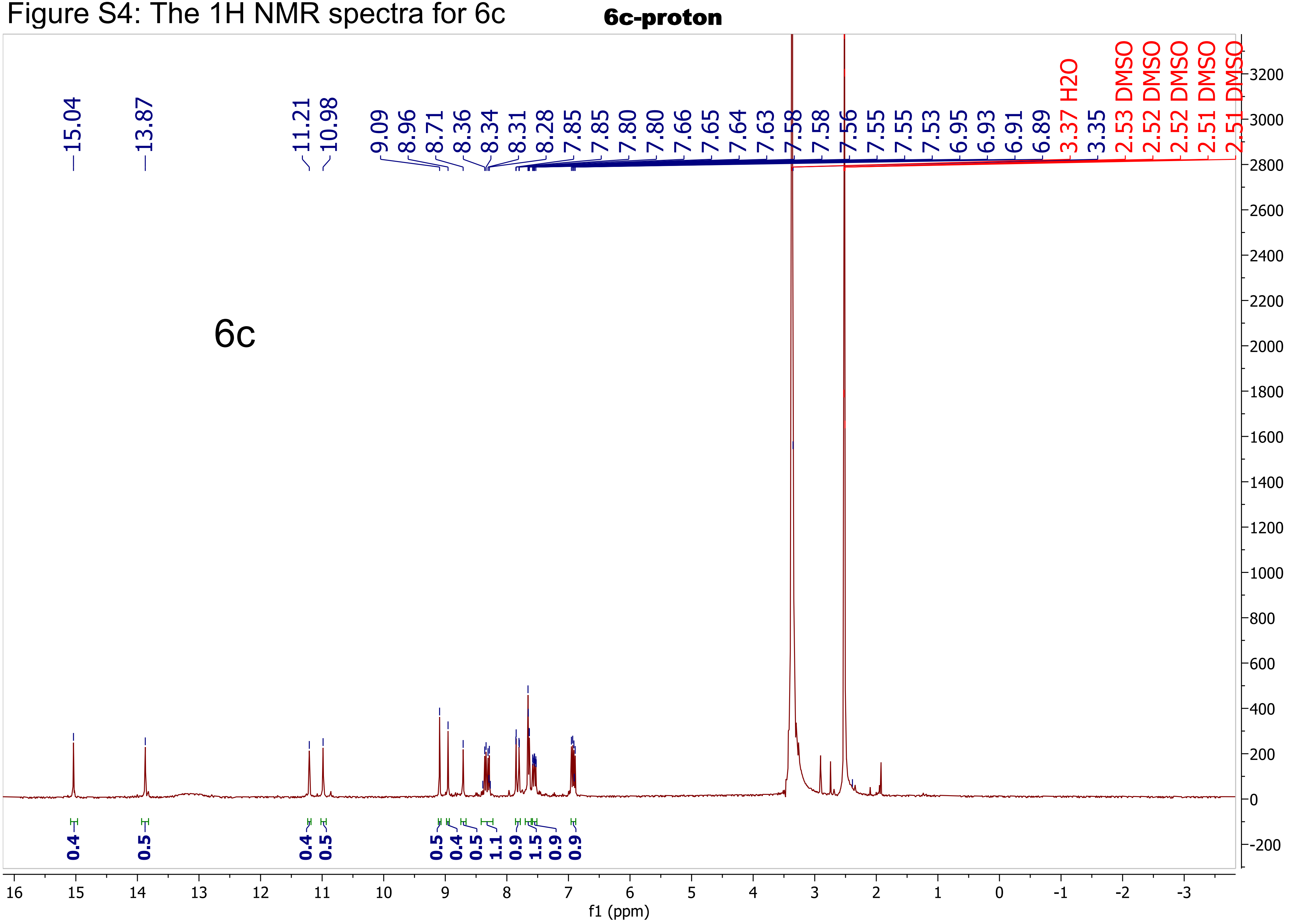

Figure S5: The 1H NMR spectra for 6d

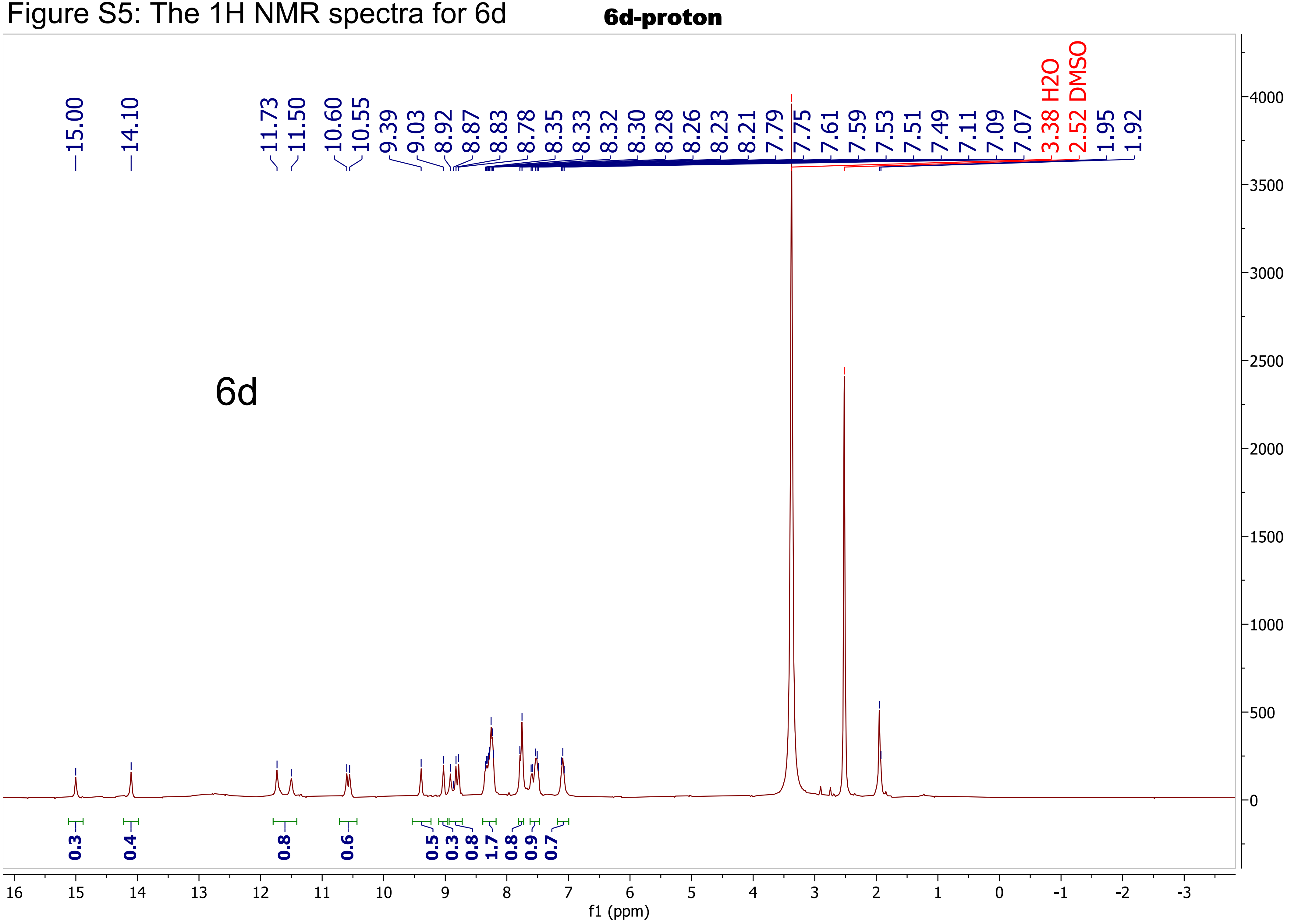

Figure S6: The 1H NMR spectra for 6e

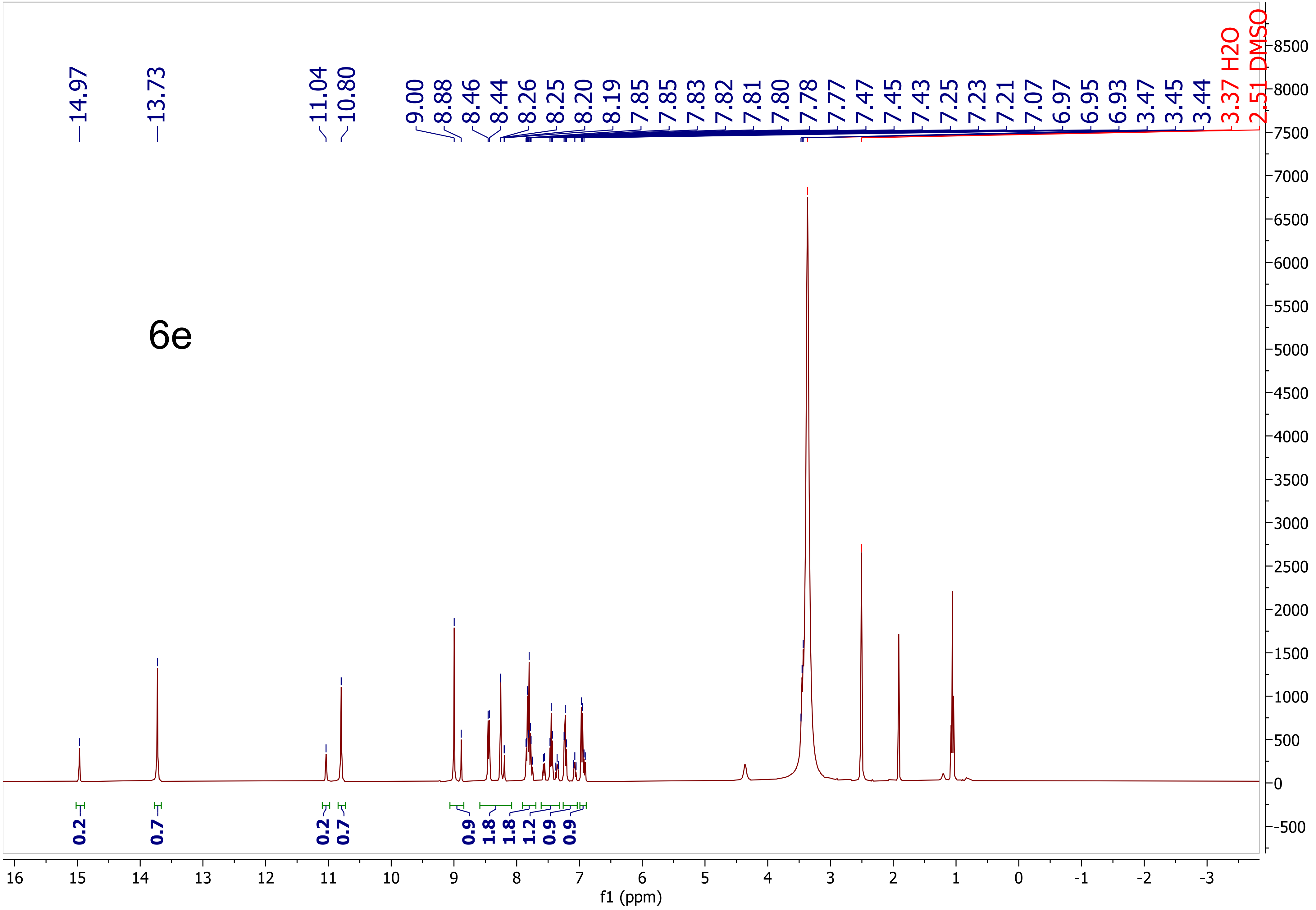

Figure S7: The 1H NMR spectra for 6f

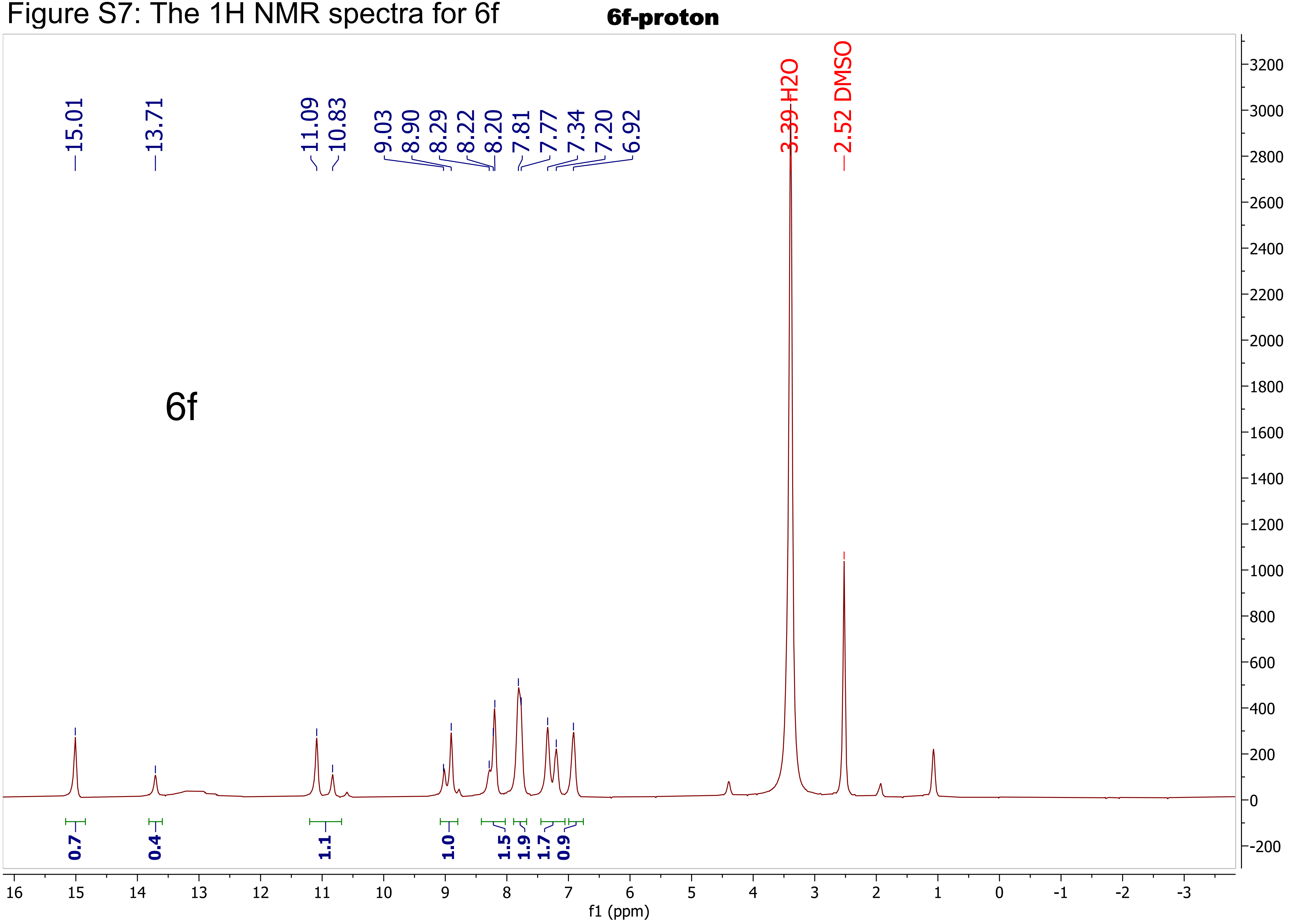

Figure S8: The 13C NMR spectra for 6f

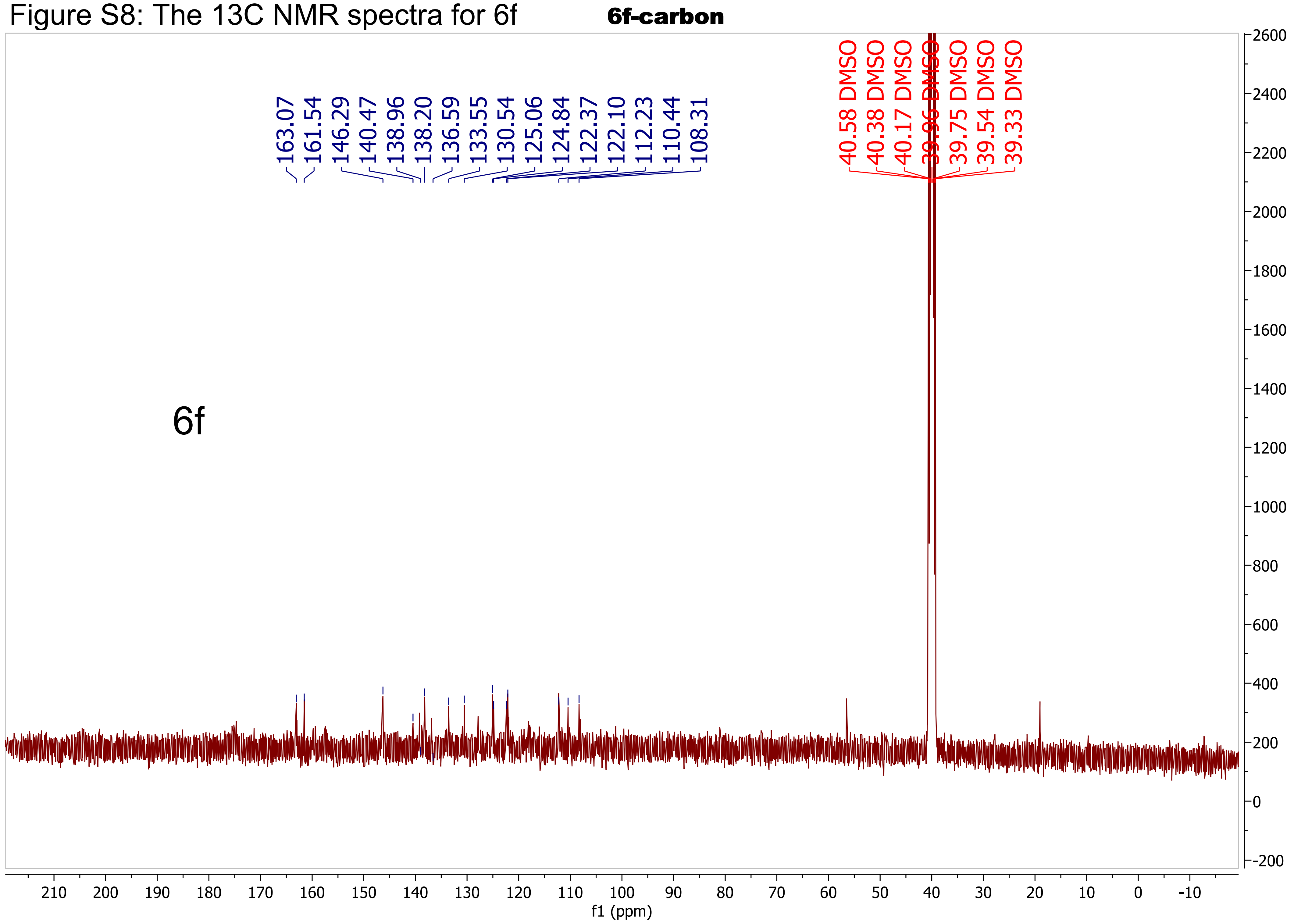

Figure S9: The 1H NMR spectra for 6g

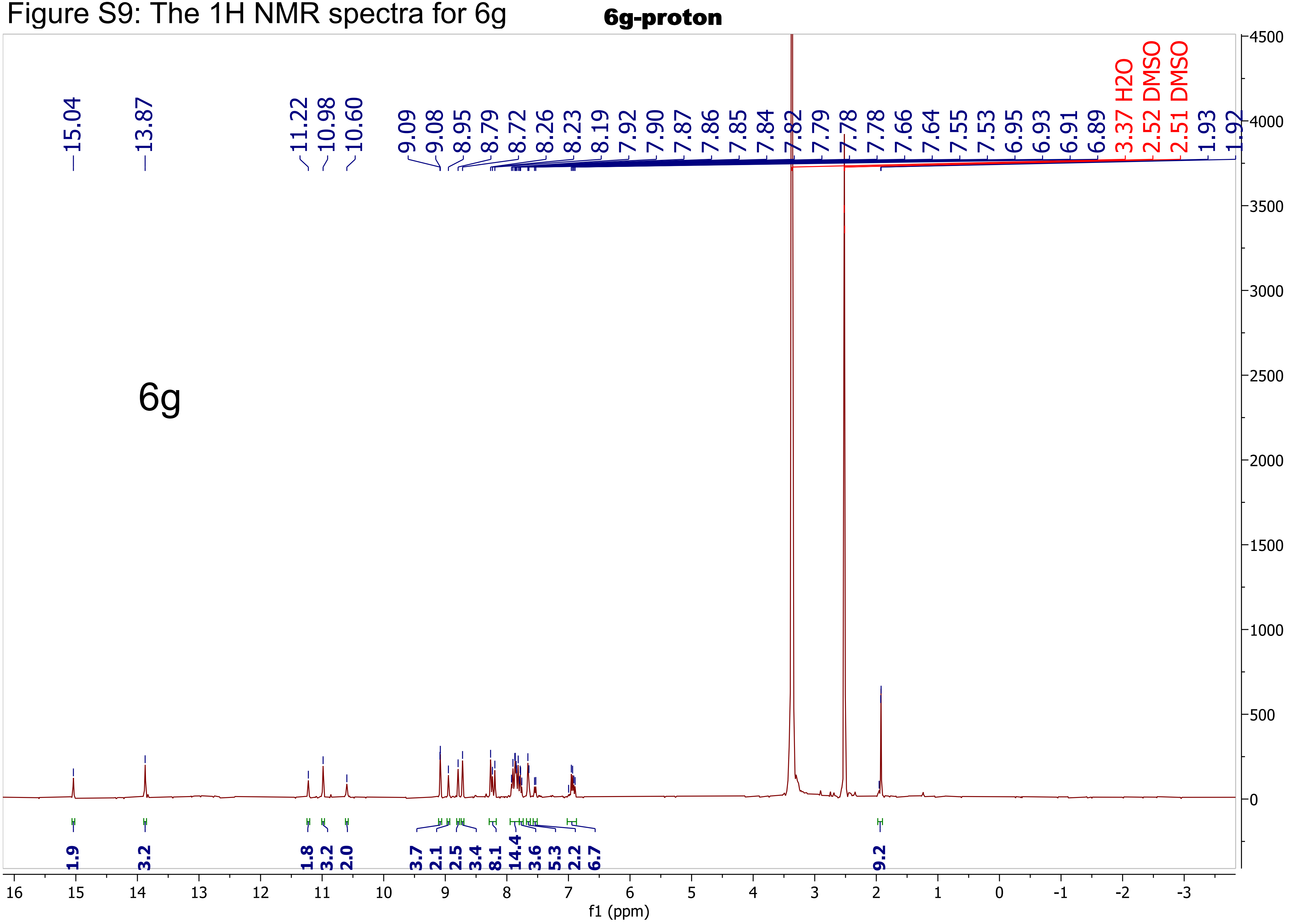

Figure S10: The 13C NMR spectra for 6g

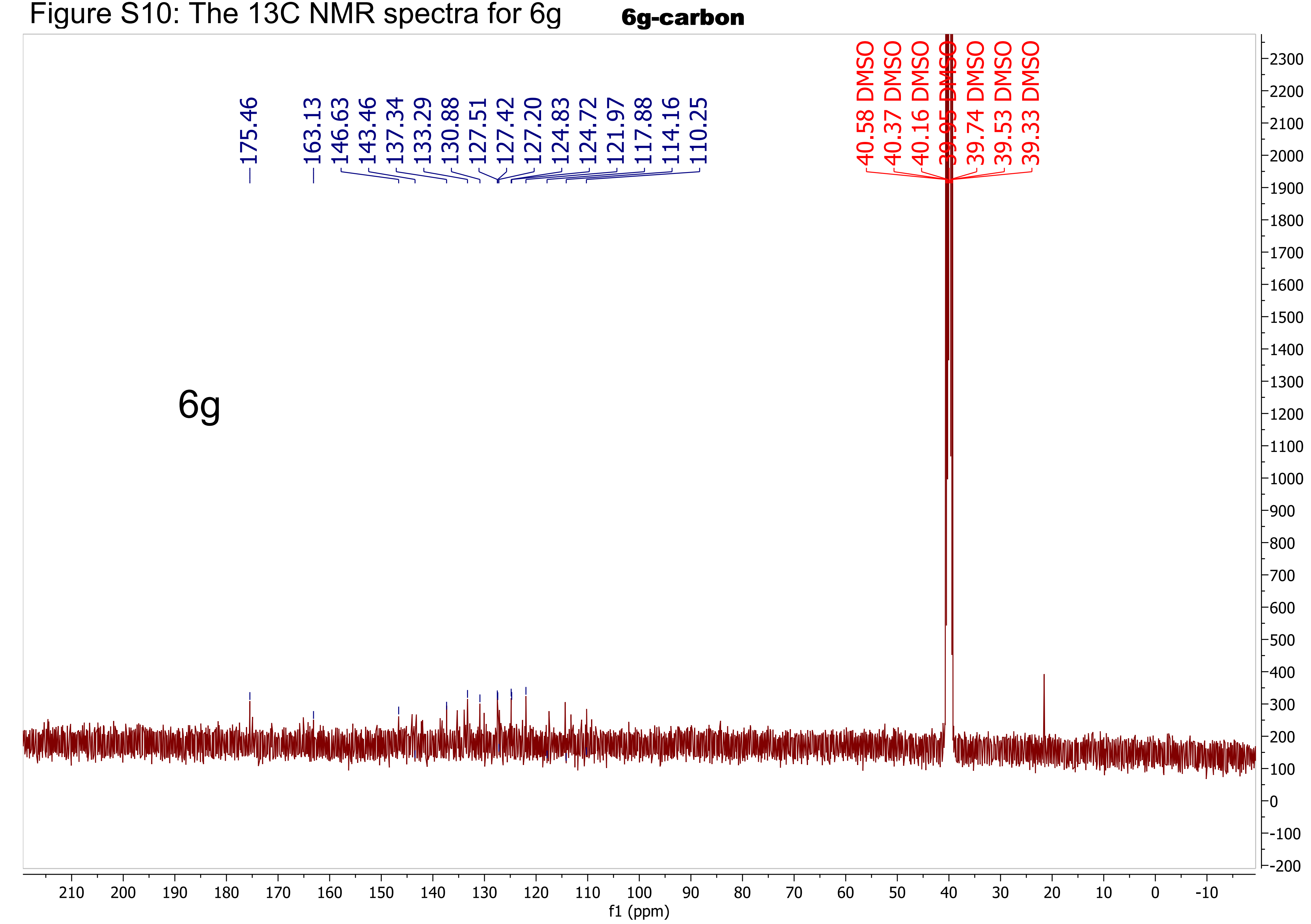

Figure S11: The 1H NMR spectra for 6h

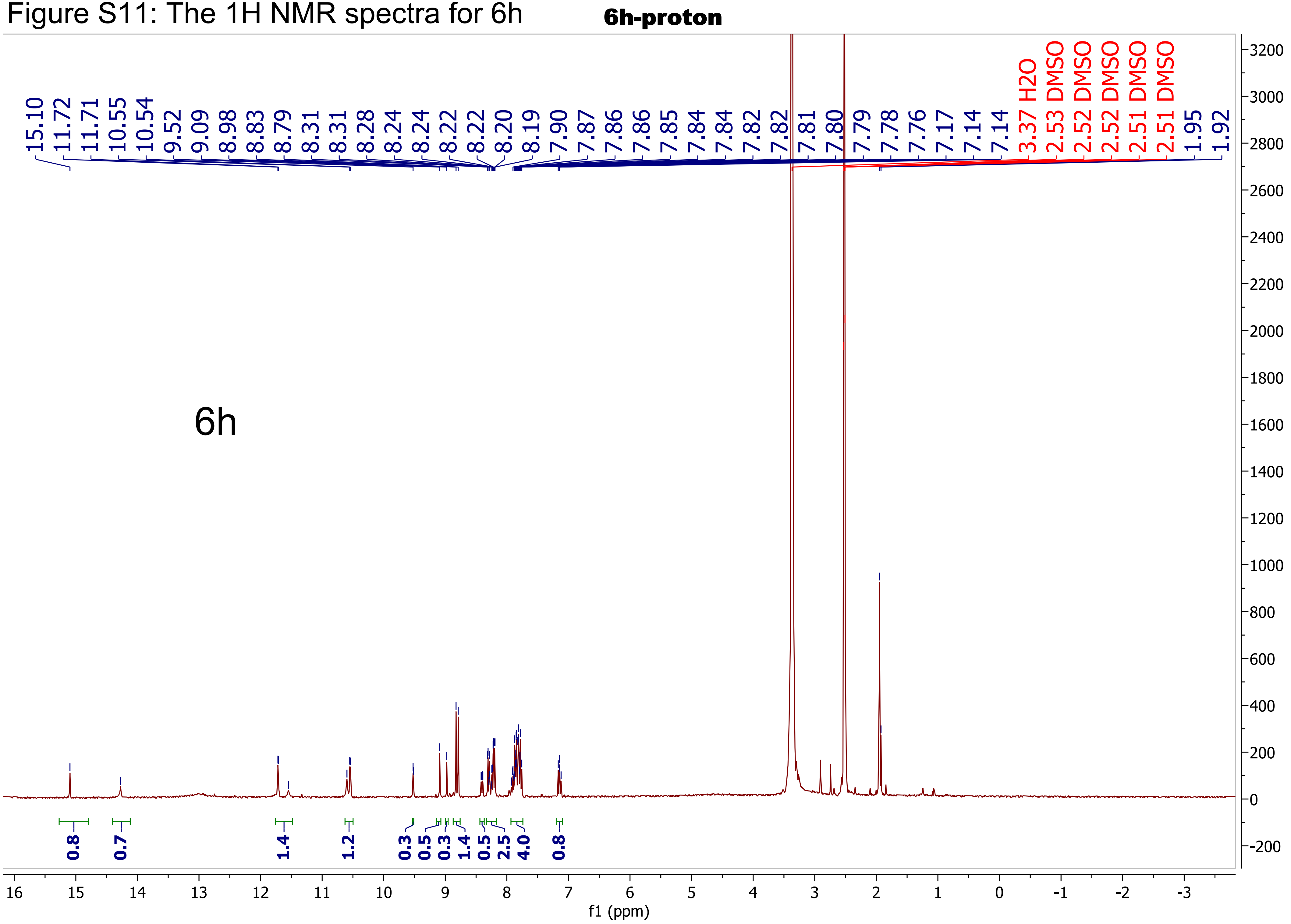

Figure S12: The 1H NMR spectra for 8a

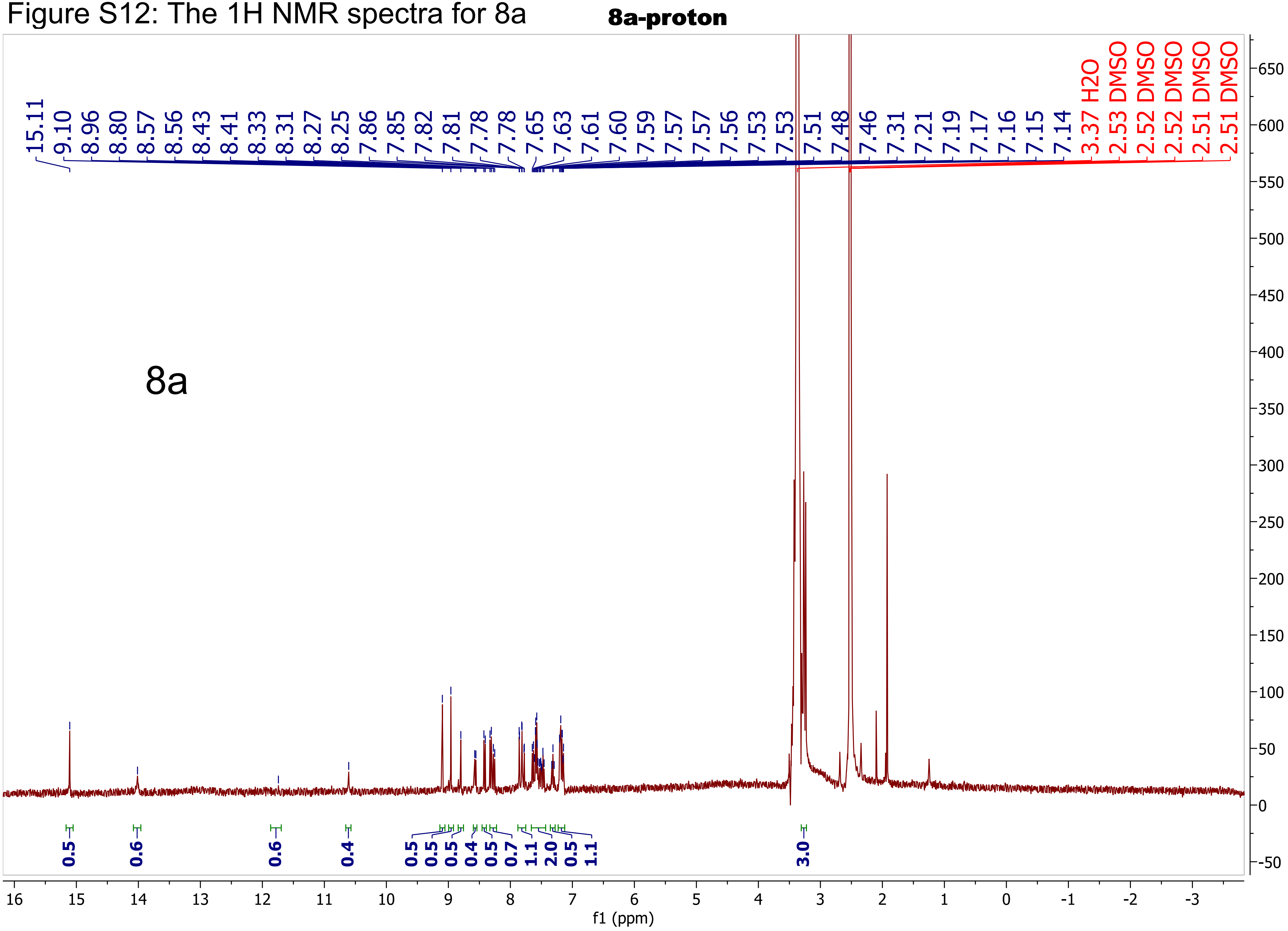

Figure S13: The 1H NMR spectra for 8b

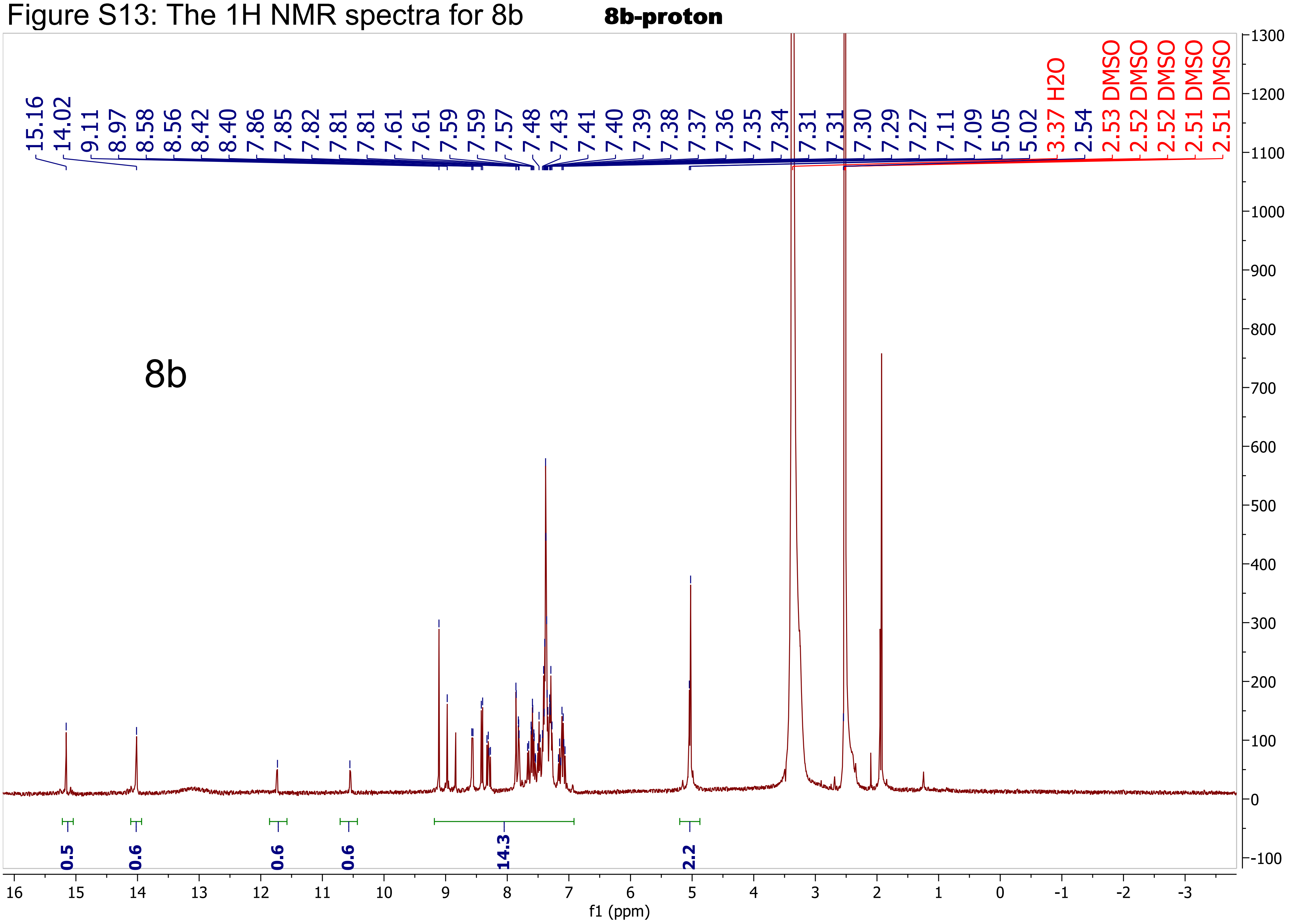

Figure S14: The 1H NMR spectra for 8c

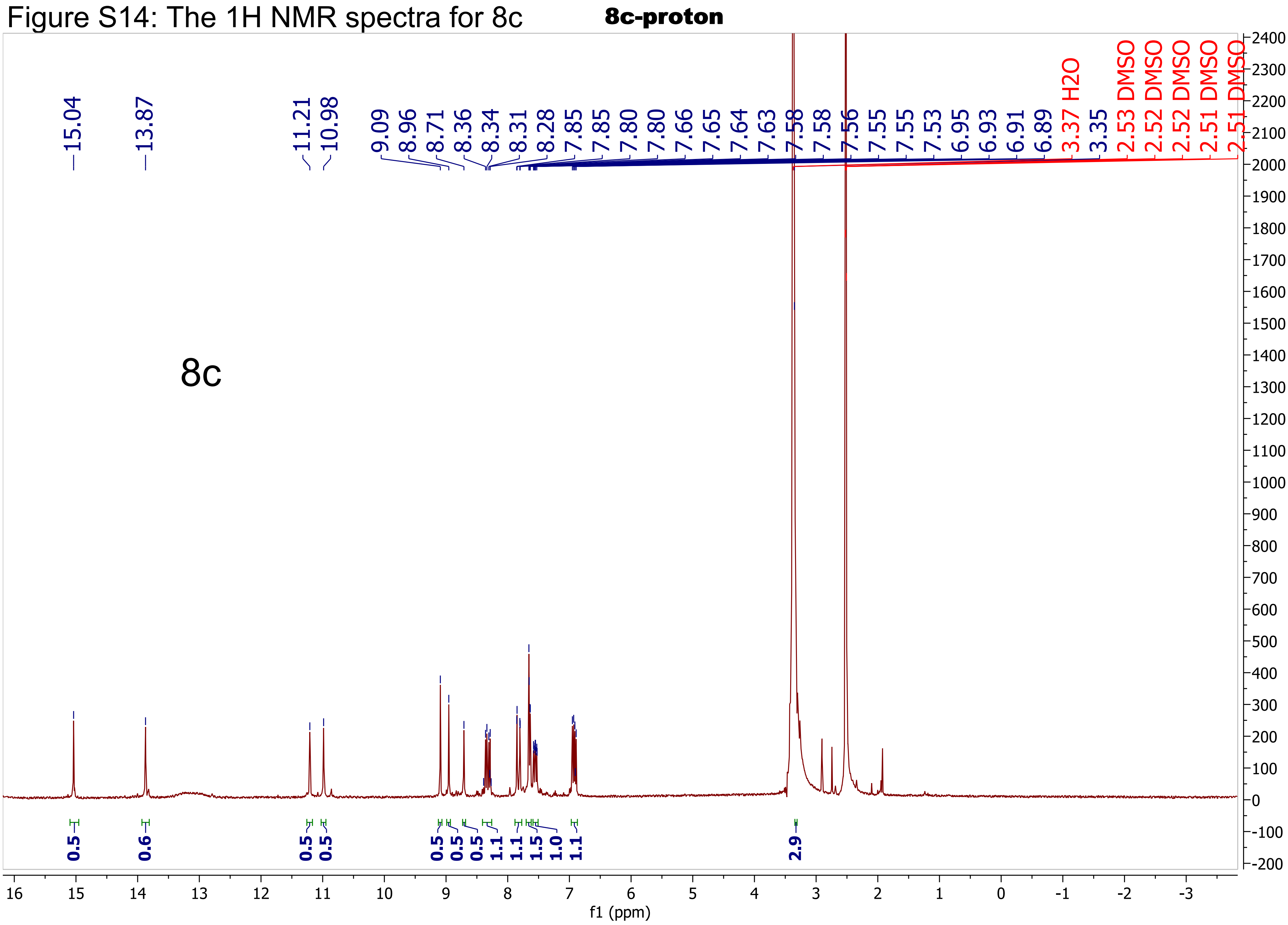

Figure S15: The 1H NMR spectra for 8d

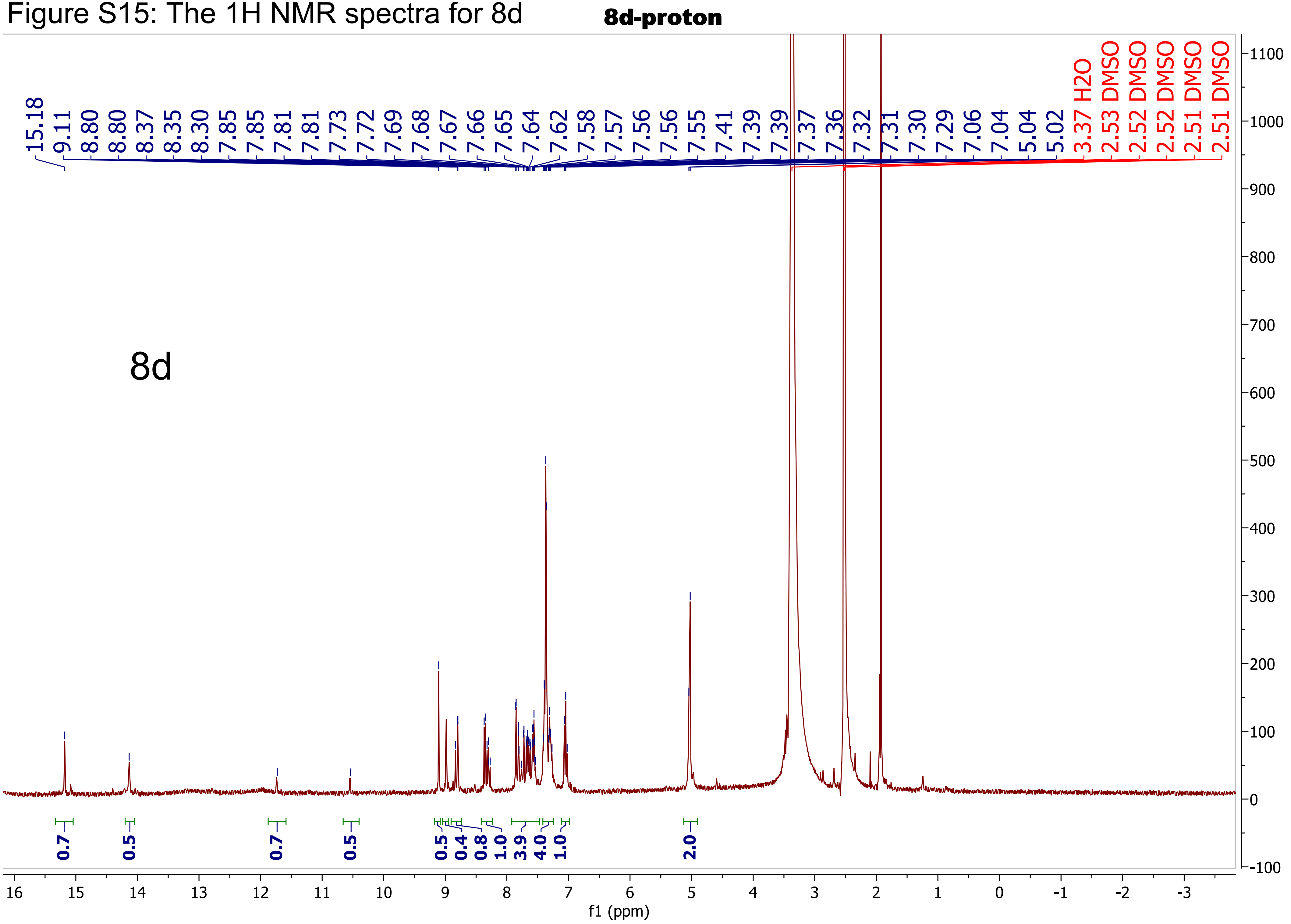

Figure S16: The 1H NMR spectra for 8e

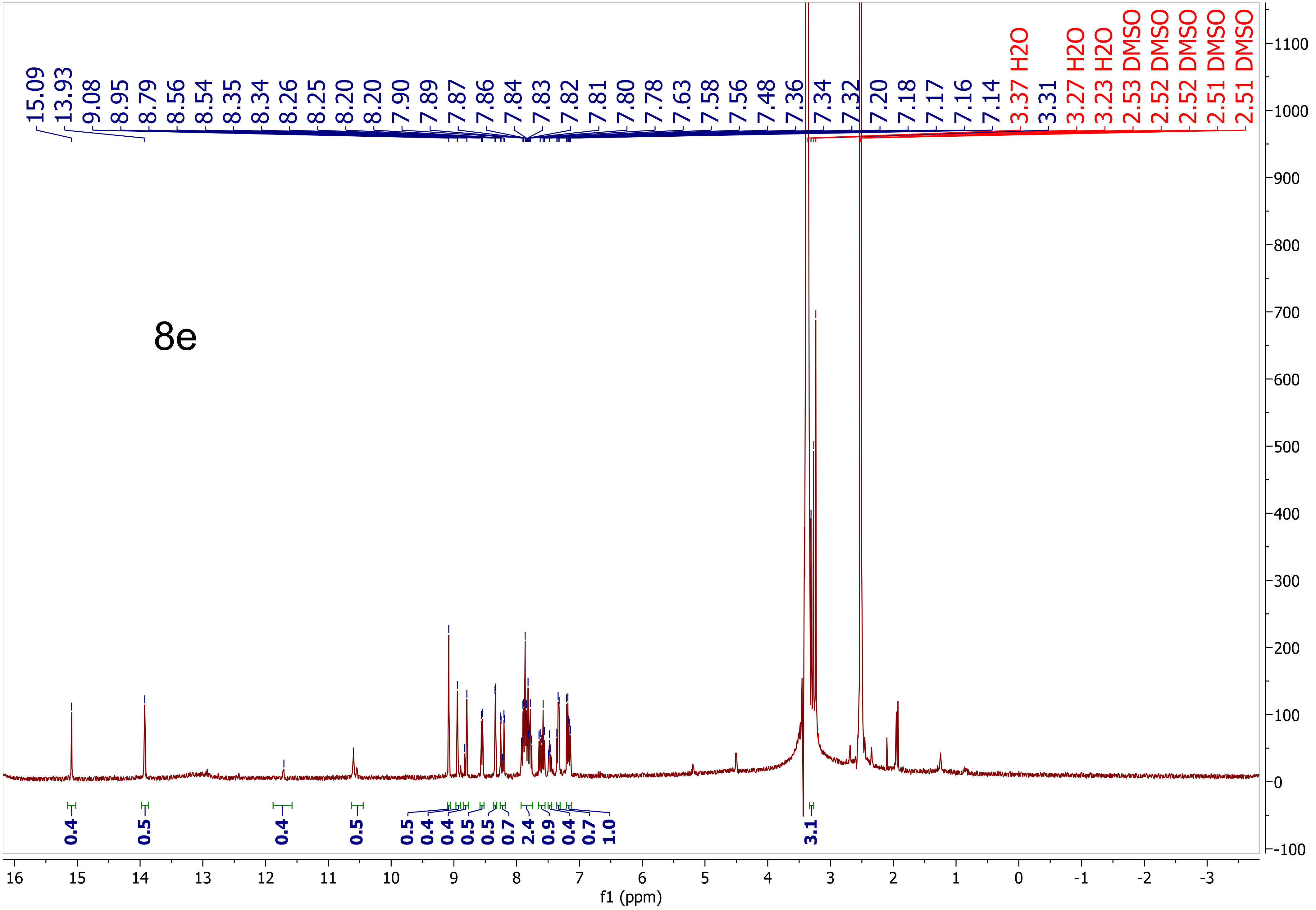

Figure S17: The 1H NMR spectra for 8f

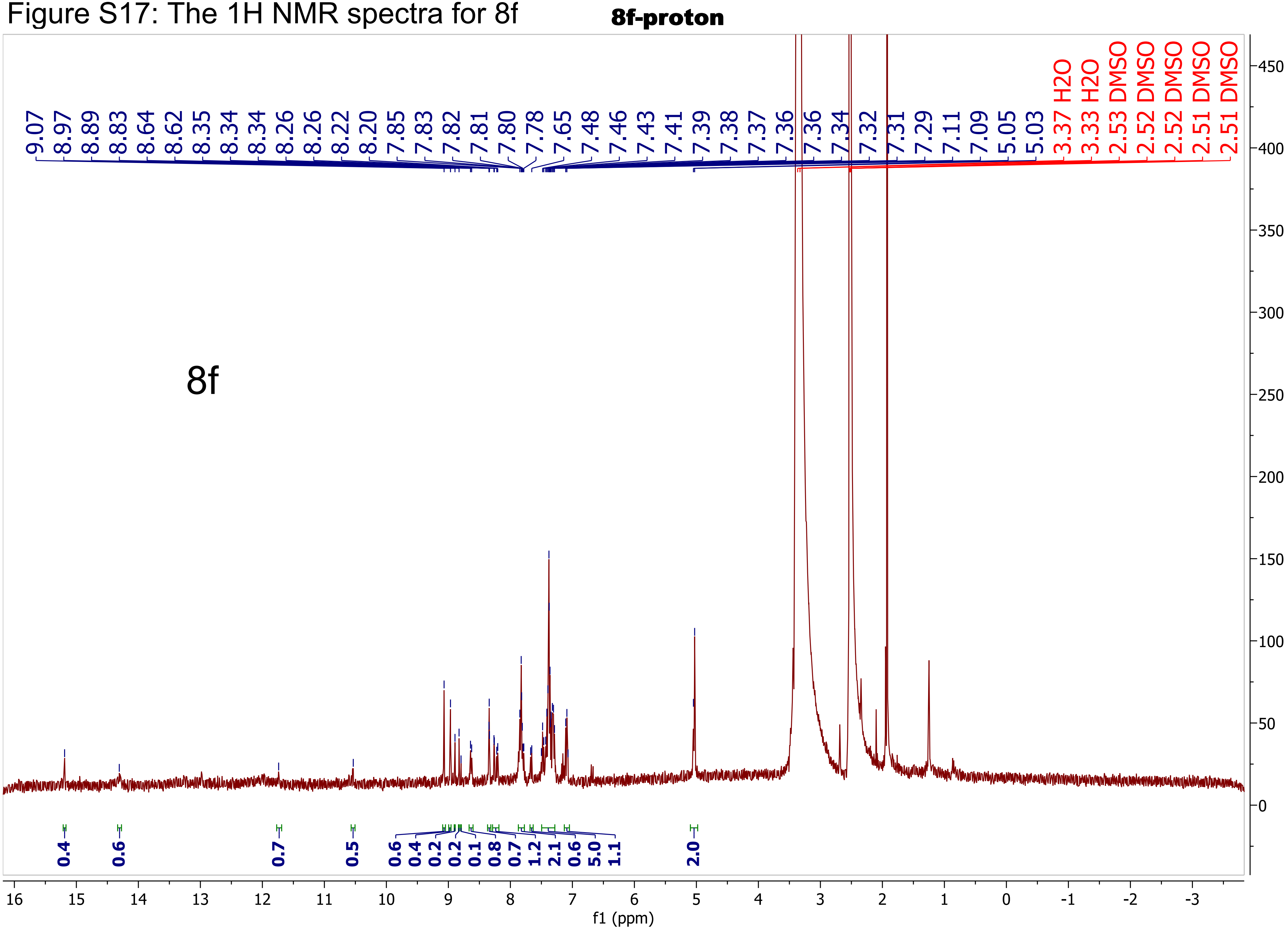

Figure S18: The 1H NMR spectra for 8g

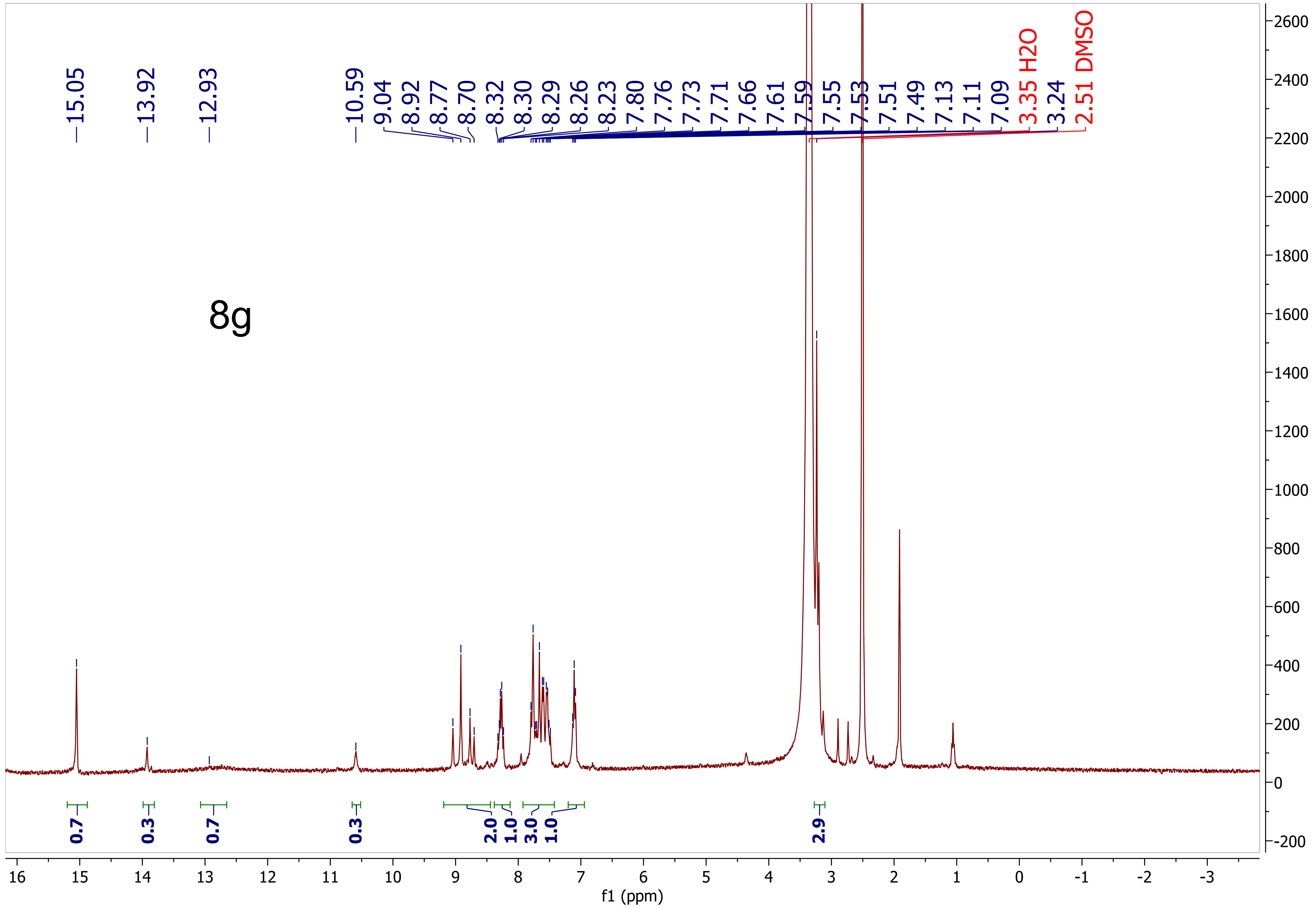

Figure S19: The 1H NMR spectra for 8h

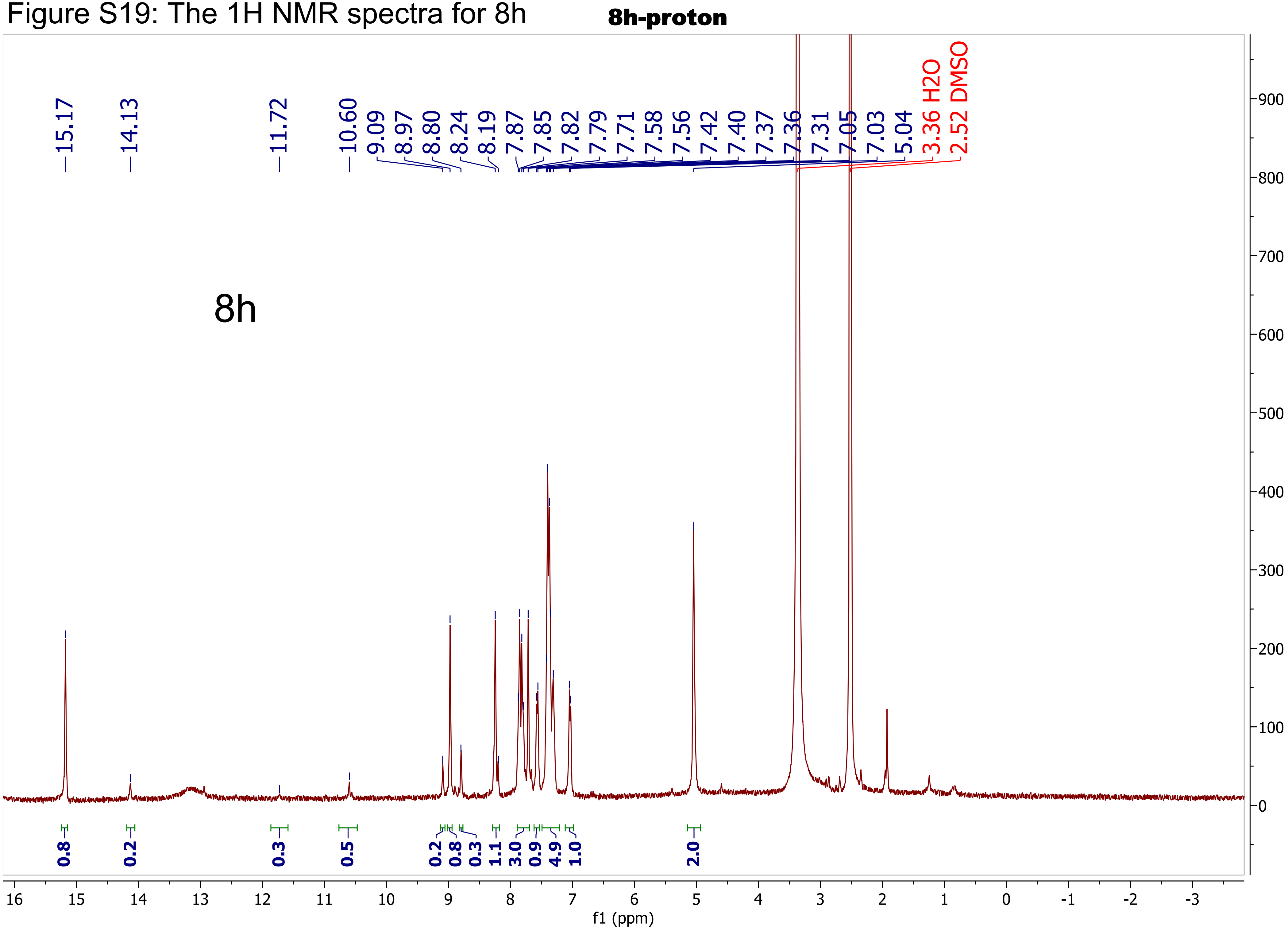

Figure S20: The IR spectra for 6a

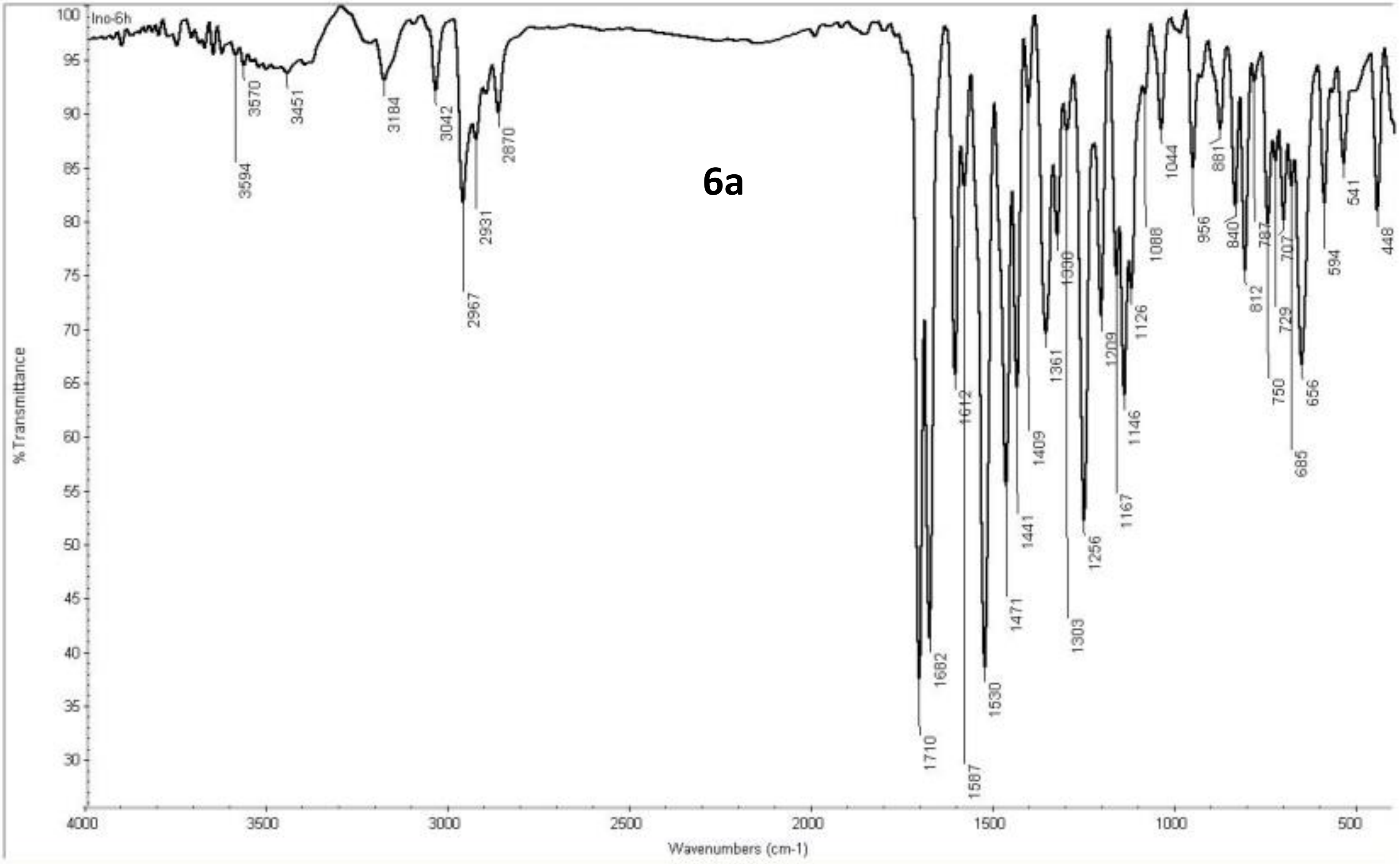

Figure S21: The IR spectra for 6d

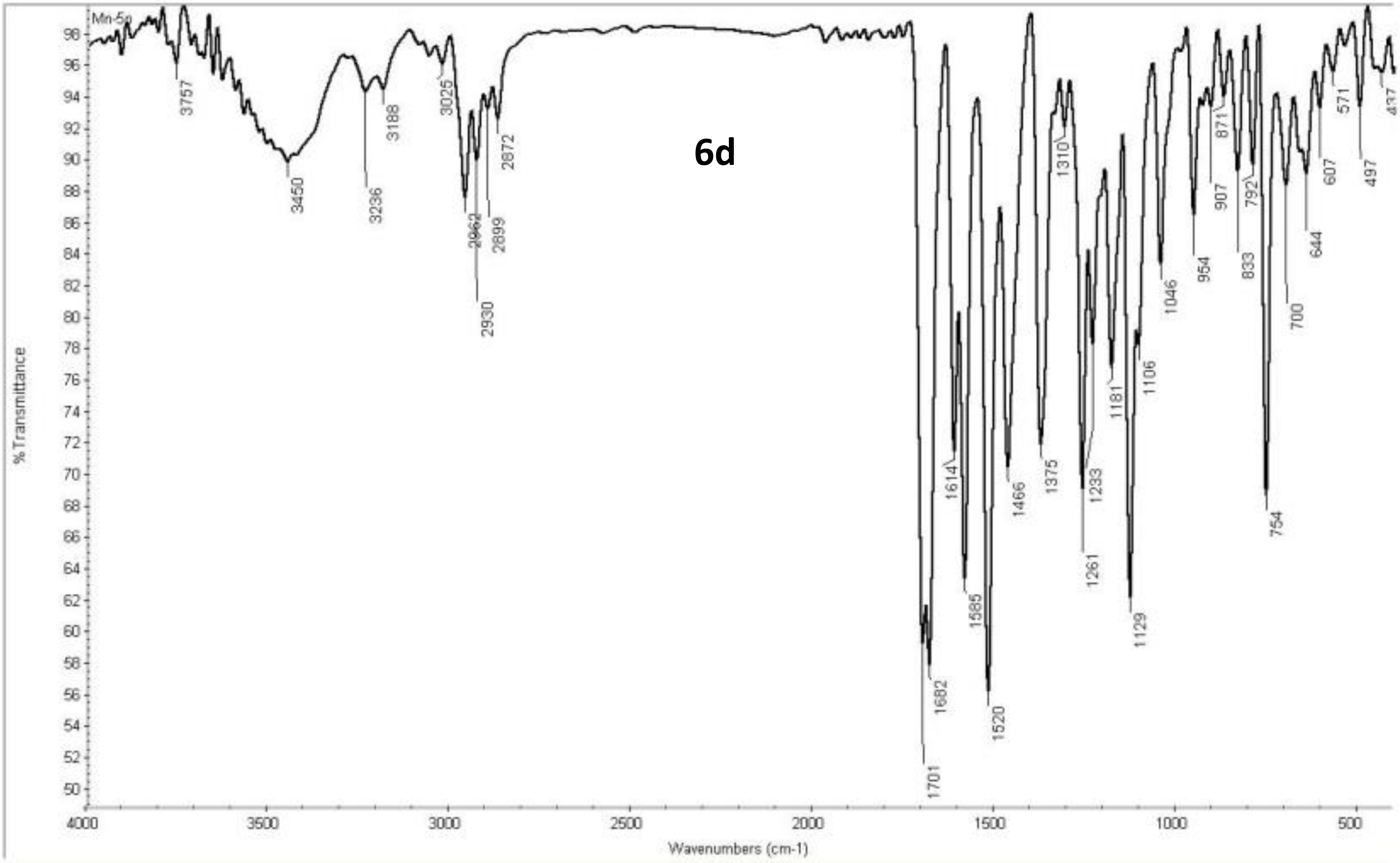

Figure S22: The IR spectra for 6g

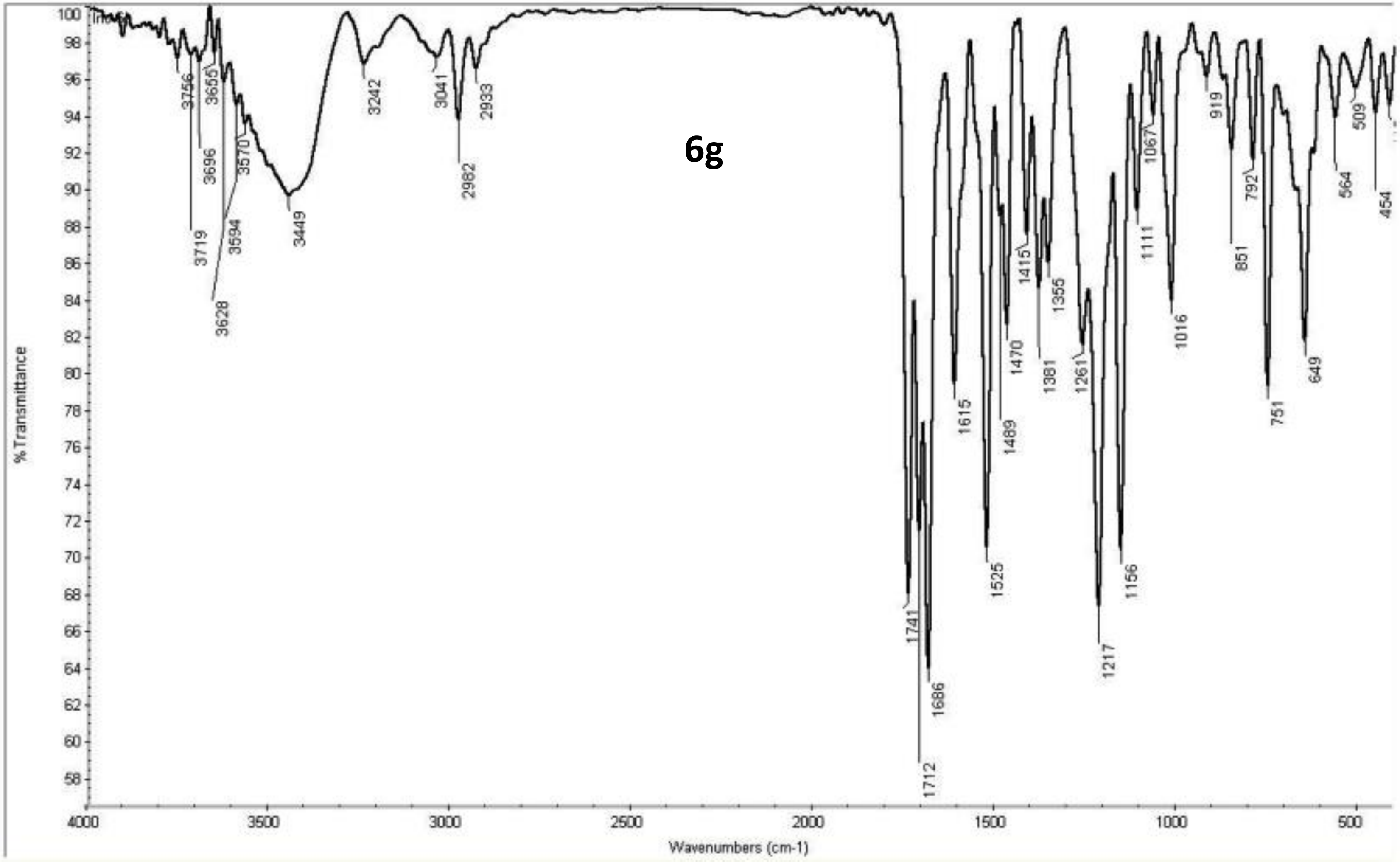

Figure S23: The IR spectra for 6h

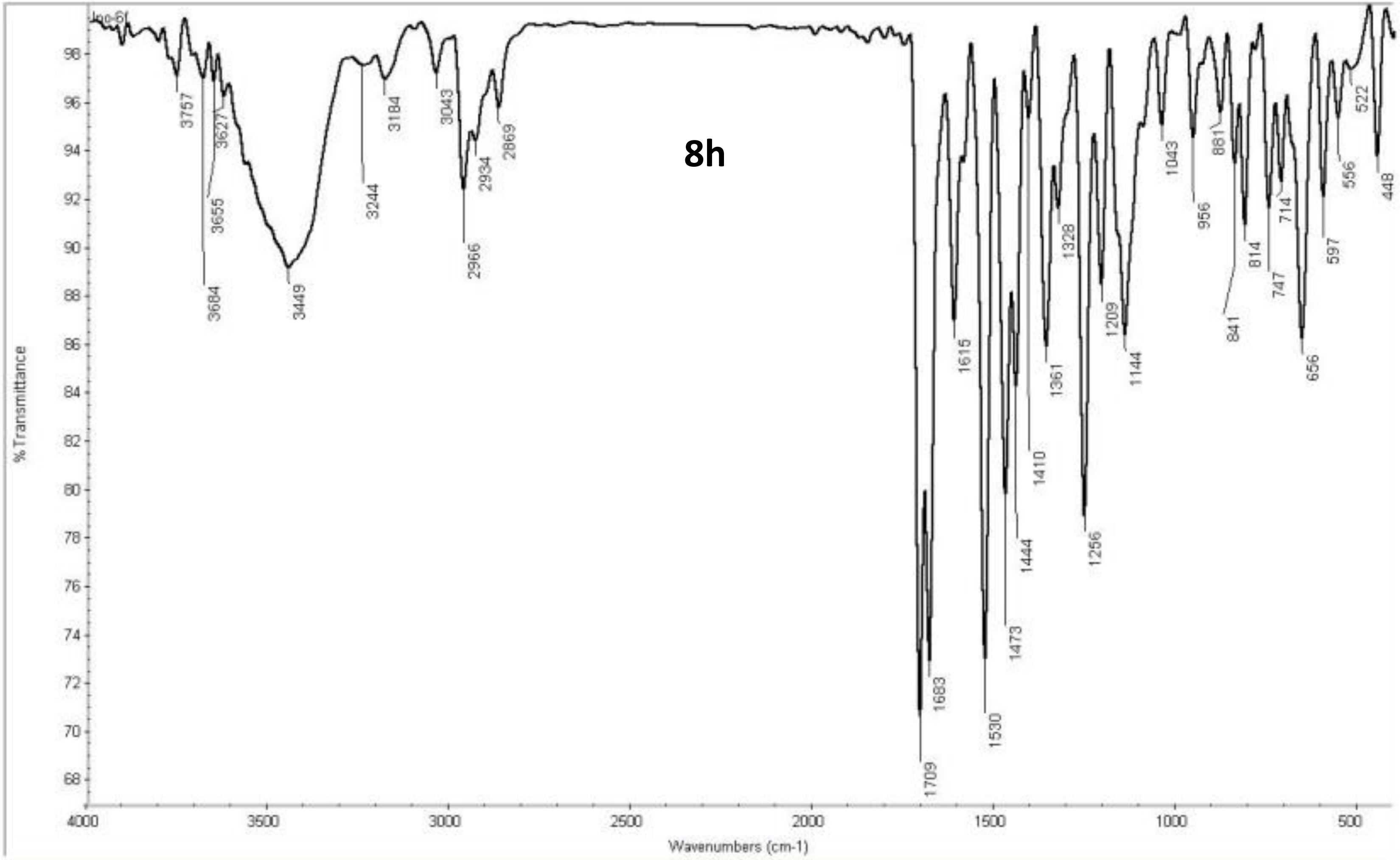

Supplement: Supplementary file 1 [file molecules-27-08807-s001.zip › molecules-2052501-supplementary.pdf]
